# Recent developments in photoredox-catalyzed remote *ortho* and *para* C–H bond functionalizations

**DOI:** 10.3762/bjoc.16.26

**Published:** 2020-02-26

**Authors:** Rafia Siddiqui, Rashid Ali

**Affiliations:** 1 Department of Chemistry, Jamia Millia Islamia, New Delhi-110025, India

**Keywords:** dual catalysis, light, *ortho* and *para* C–H bond functionalization, photoredox catalysis

## Abstract

In recent years, the research area of direct C–H bond functionalizations was growing exponentially not only due to the ubiquity of inert C–H bonds in diverse organic compounds, including bioactive natural and nonnatural products, but also due to its impact on the discovery of pharmaceutical candidates and the total synthesis of intricate natural products. On the other hand, more recently, the field of photoredox catalysis has become an indispensable and unparalleled research topic in modern synthetic organic chemistry for the constructions of challenging bonds, having the foremost scope in academia, pharmacy, and industry. Therefore, the development of green, simpler, and effective methodologies to accomplish direct C–H bond functionalization is well overdue and highly desirable to the scientific community. In this review, we mainly highlight the impact on, and the utility of, photoredox catalysts in inert *ortho* and *para* C–H bond functionalizations. Although a surge of research papers, including reviews, demonstrating C–H functionalizations have been published in this vital area of research, to our best knowledge, this is the first review that focuses on *ortho* and *para* C–H functionalizations by photoredox catalysis to provide atom- and step-economic organic transformations. We are certain that this review will act as a promoter to highlight the application of photoredox catalysts for the functionalization of inert bonds in the domain of synthetic organic chemistry.

## Introduction

Over a short period of time, direct C–H bond functionalizations by photoredox catalysts have become a preferred research area for the scientific community. Although a number of reviews already appeared in the literature regarding C–H bond functionalizations, in this review, we are purely focusing on the pros and contras of photoredox catalysts over other catalysts for inert C–H bond functionalizations. Herein, we will broadly discuss the different catalytic systems that facilitate *ortho* and *para* C–H functionalization by utilization of effective and feasible photoredox catalysts (with the aid of transition metals), hydrogen atom transfer, and aerobic oxidation.

**Figure 1:**
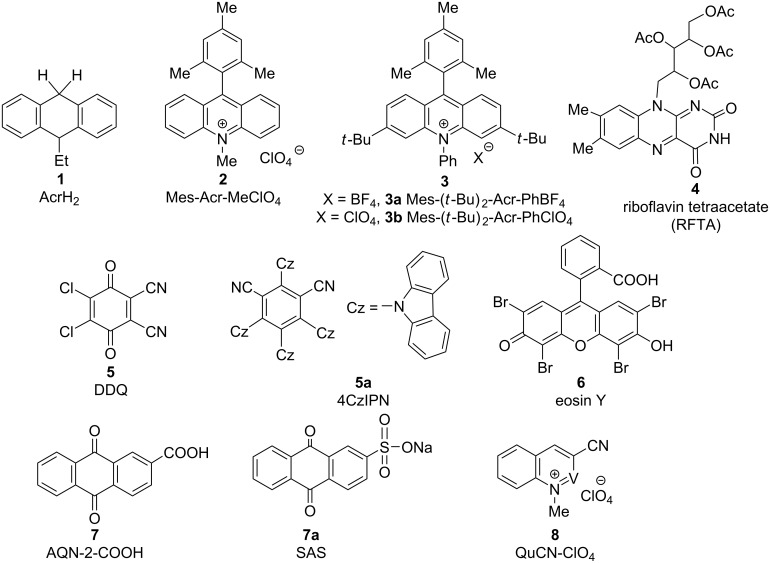
List of photoredox catalysts used for C–H bond functionalizations.

**Figure 2:**
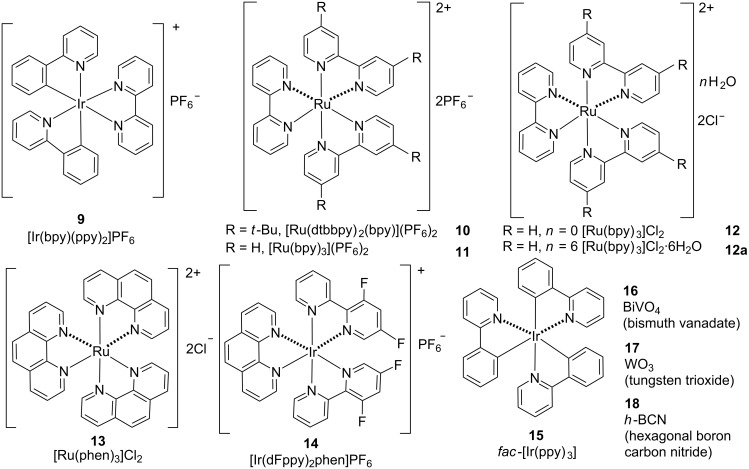
List of metal-based photoredox catalysts used in this review article.

Over the last two decades, direct C–H bond functionalization, which has historically been difficult to perform, was a much-awaited and demanding method in the field of modern synthetic chemistry [1–14]. As can be seen in the literature, most of the earlier work regarding C–H bond functionalization has been done either using transition metal catalysis or organocatalysis, through the installation of directing groups next to the targeted C–H bond, or by employing radical tactics based on single-electron transfer (SET) [15–27]. Although groundbreaking advancements were accomplished in this wonderful area of research, transforming a specific C–H bond effectively and selectively under favorable conditions (viz, room temperature, without external oxidant, cost-effective, sustainable, and environmentally friendly) still remains a highly challenging issue to the scientific community. In this context, visible light-induced photoredox catalysis, which is thought to be an abundant, inexpensive, renewable, and nonpolluting chemical transformation, has attracted increasing attention during the past years due to the extraordinary competence and exceptional reactivity pattern of the method [28–29]. Although over the past few decades, photoredox catalysis found diverse applications, ranging from material and environmental sciences to biomedical sciences, the involvement in synthetic organic chemistry, in particular C–H bond functionalization, is still in its infancy. Nowadays, photoredox catalysis is on the forefront as a potent strategy for bond modifications through multicatalytic strategies and the invention of nontraditional methodologies. It is enormously effective in the generation of radicals by manipulating the transition metal complexes and organic dyes involved [30]. Inspiring work by Pac, Kellog, Deronzier, and Sauvage on novel organic syntheses were focused on radical generation via photoredox catalysis, which has advanced C–H functionalizations to a higher level [31–34]. However, a major contribution to the applications of photoredox catalysis in organic synthesis has been done by Sanford, MacMillan, Glorius, Rueping, Molander, etc. [35–40]. Inert C–H bond functionalizations via photoredox catalysis impart the best alternative not only to earlier reported approaches but also to build new C–X (X = C, B, N, O, S) bonds for architecturally simple, yet challenging molecules, which are otherwise highly difficult or impossible to be formed by other methods. These practices count on the competence of metal complexes and organic dyes to convert visible light into chemical energy via SET events, providing a simplistic access to open-shell intermediates. These two synchronizing and rapidly flourishing areas of research in modern synthetic chemistry have recently been merged, and their association was found to be very effective in terms of chemo-, regio-, and stereoselectivity aspects. Therefore, we believe that a comprehensive review in this field of research, i.e., the applications of photoredox catalysts for the functionalization of inert C–H bonds, is well overdue and highly desirable to provide a new dimension to the associated synthetic organic chemistry. Although there are plenty of separate reviews on C–H bond functionalization and applications of photoredox catalysis in organic chemistry, herein, we cover examples that only involve photoredox catalysts for selective *ortho* and *para* C–H bond functionalization. A list of photoredox catalysts employed in this review is shown in Figure 1 and Figure 2.

**Figure 3:**
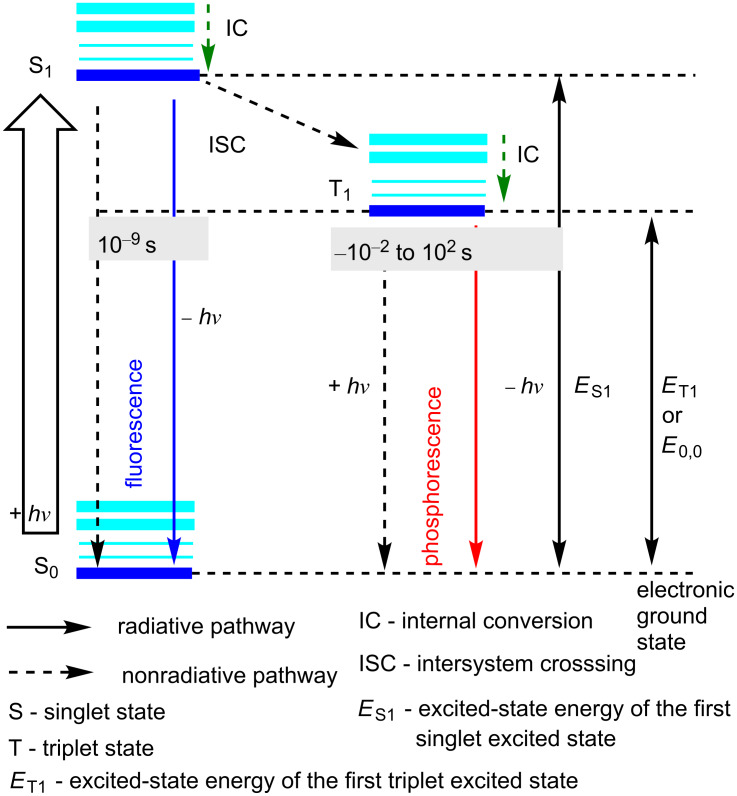
Jablonski diagram.

The most frequently used mechanisms of converting light energy into chemical energy using photoredox catalysts are: (i) photoredox catalysis; (ii) organometallic excitation; (iii) light-induced atom transfer, and (iv) energy transfer. Basically, a photoredox catalyst transforms light energy into chemical energy via the generation of reactive intermediates through electron transfer reactions. A photochemical reaction is directed by the photophysical properties of an electronically excited molecule. The first vibrational equilibrated singlet excited state is S_1_, and it depends on both nonradiative and radiative pathways. Light is emitted (− *hv*) upon modulation of radiative pathways to lower energy states, whereas in nonradiative pathways, dissipated energy is lost in the form of heat. In the case of fluorescence (a radiative pathway with a short lifetime), S_1_ returns to S_0_. On the other hand, S_1_ can also transition to the spin-forbidden T_1_ through intersystem crossing (ISC), which then decays by (longer) radiative processes to S_0_ (phosphorescence. Figure 3) [41–43]. The triplet excited state is oxygen-sensitive as O_2_ carries out quenching in solution, thereby leading to the disappearance of phosphorescence, following the Frank–Condon principle [44–45]. As far as the singlet and triplet excited states of organic dyes and organometallic compounds are concerned, there is a difference in lifetimes. Usually, the lifetime of excited states of organic dyes is nanoseconds, whereas for organometallic compound, the time frame is microseconds. In fact, it has been observed that by virtue of multiple symmetry-allowed and -forbidden electronic transitions, the exact scenario of excited states is highly complex, and thereby offering diverse multiplicities to ground and excited states [46–48].

**Figure 4:**
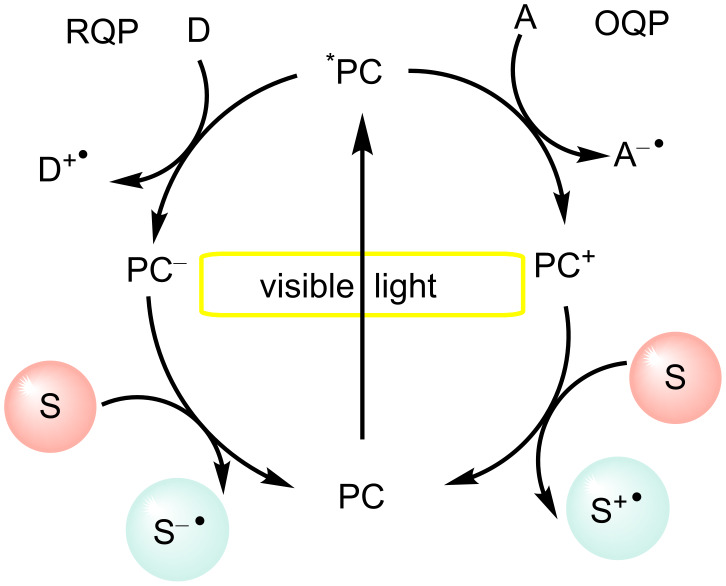
Photoredox catalysis via reductive or oxidative pathways. D = donor, A = acceptor, S = substrate, PC = photoredox catalyst, RDP = reductive quenching pathway, OQP = oxidative quenching pathway.

In photoredox catalysis, visible light gets absorbed by the photocatalyst (PC), which transitions into a photoexcited state (*PC) that can undergo either energy transfer or redox pathways. As can be seen in Figure 4, the redox pathway consists of reductive and oxidative quenching pathways. Furthermore, it has also been observed that the excited-state species are more oxidizing or reducing than the species in the ground state. This is due to the availability of the half-empty low-energy orbital and the presence of an electron in a high-energy orbital, respectively [49–50].

## Review

### Why photoredox catalysis?

Over the past century, the discovery, development, and application of light-dependent catalysts have permitted the invention of a range of nontraditional bond formations in the realm of synthetic organic chemistry. In recent years, the research area of photoredox catalysis has experienced a noteworthy renaissance not only because of the lower toxicity, stability, speed, and efficiency of the method, but also thanks to the easy generation of radical species and the formation of a long-lived triplet excited state under photoirradiation that can behave as a reductant as well as an oxidant [51–52]. Under normal conditions, most organic molecules do not absorb UV light of high intensity, confining the capacities of conventional reactions [53]. The photoredox catalysts, apart from being recoverable and reusable, have several advantages when compared to the traditional radical pathway and transition metal catalysis, which include: (i) excellent regioselectivity of the targeted C–H bond formations thanks to favorable dissociation enthalpies and electronic properties as compared to other concurrent C–H bonds; (ii) avoidance of an extra oxidant because the reaction proceeds with overall redox neutrality; (iii) the use of household bulbs or LEDs as light sources under operationally simple reaction conditions; (iv) the high redox potential of photocatalysts that can manipulate the oxidation states of transition metal catalysts [54–55]. They have also found applications in novel solar cell functional materials [56], the reduction of carbon dioxide [57–59], etc. Additionally, inorganic chemists have utilized inorganic photocatalysts for functional components, viz, doping [60–67], encapsulated guests [68–74], molecular machines [75–78], for light harvesting, etc. [79].

**Figure 5:**
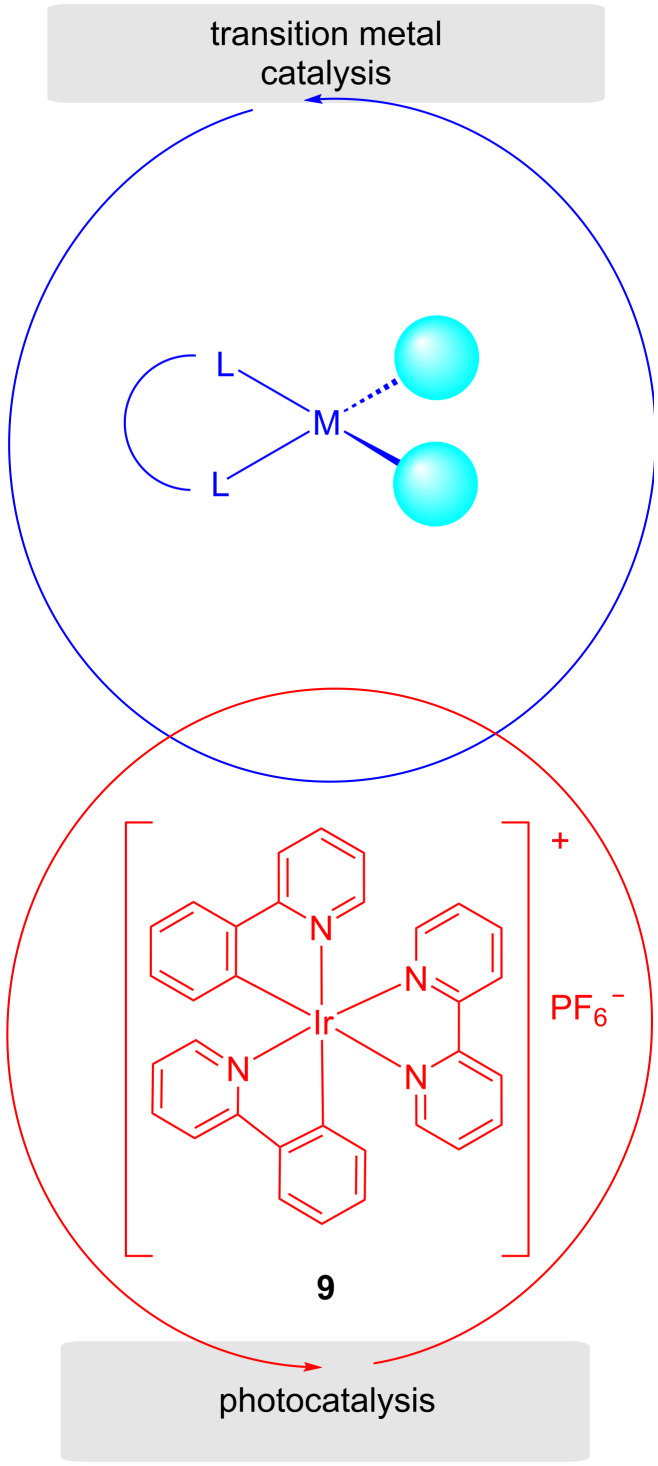
Schematic representation of the combination of photoredox catalysis and transition metal catalysis.

On the other hand, in metallaphotocatalysis (dual catalytic systems), it has been observed that the efficiency of transition metal catalysts can be enhanced with photoredox catalysts, viz, functioning under mild reaction conditions, opening of new activation modes, and undergoing synthetic transformations regio- and enantioselectively (Figure 5) [53–54]. With the aid of photoredox catalysis, the active radical moieties can easily undergo nonclassical nucleophilic direct coupling reactions. Also, there is a possibility of intermediate catalytic moiety excitation via electron transfer and modification of the oxidation state of the transition metal complexes. Such systems can be combined with different metals, for example, Ni, Co, Cu, Ru, Ir, etc. However, unexpectedly, copper is less toxic and can be utilized to catalyze reactions without the requirement of a ligand. This system is being used in C–C, C–N, C–O, C–S, and C–X bond-breaking and -forming processes [55].

Similarly, photoinduced direct hydrogen atom transfer (HAT) catalysis also plays a major role in the functionalization of intricate molecules. Photocatalysts that can undergo this process are uranyl cations, polyoxometallates, and benzophenones [9,80], but a major drawback is the limited availability of photocatalysts that can perform direct HAT. Therefore, there is a high demand for a direct-HAT catalyst that is accessible, metal-free, allows no side reactions, and can be activated by visible light easily. Recently, aerobic oxidation with visible light and photoredox catalysts has also gained a lot of attention in the modification and generation of new C–H functionalization methodologies [81–82]. The above-mentioned advantages make the photoredox processes a versatile tool in diverse fields, and we hope that these findings may act as a catalyst to boost the application of photoredox catalysis in contemporary organic synthesis.

### Aryl *ortho* C–H bond functionalization

As far as C–H bond functionalizations and photoredox catalysis are concerned, there are limited reports available in the literature describing *ortho* C–H bond functionalizations using photoredox catalysis. To our best knowledge, this is the first review that merges these two coexisting and fast-growing areas of research. Herein, we have tried to assemble all reported methods for the selective functionalization of aryl *ortho* C–H bonds.

#### C–H olefination

**Scheme 1:**
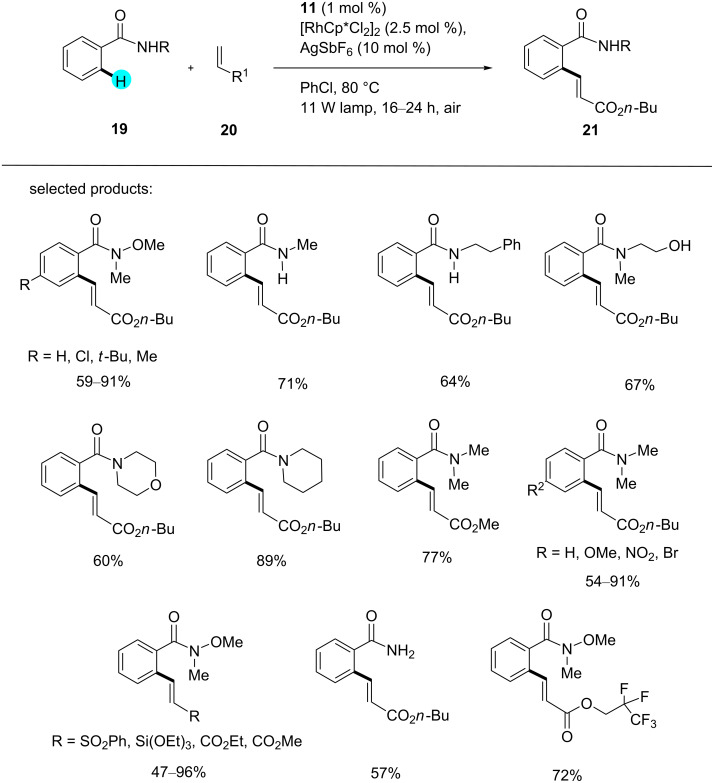
Weinreb amide C–H olefination.

**Figure 6:**
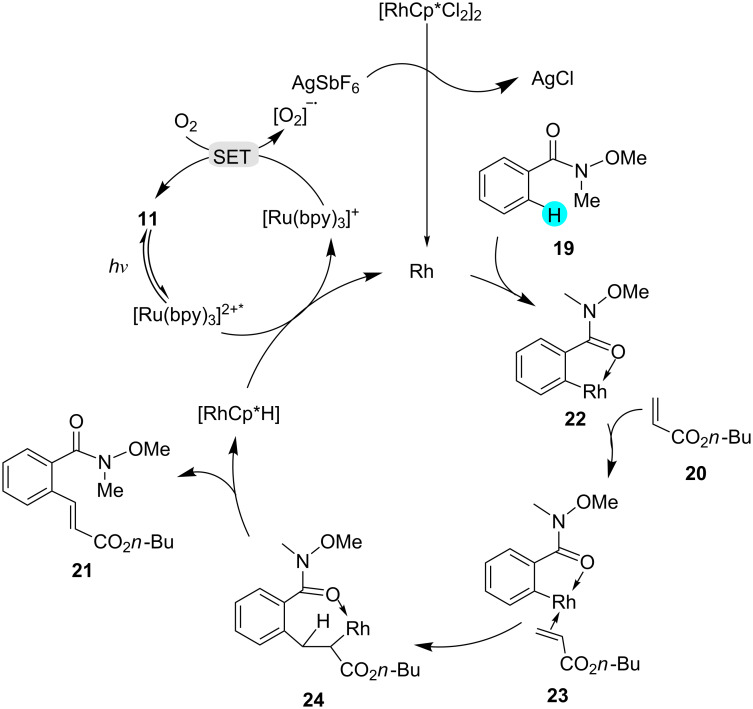
Mechanism for the formation of **21** from **19** using photoredox catalyst **11**.

**Weinreb amide C–H olefination:** Photoredox catalysts carry the reactions forward with high regioselectivity, good functional group tolerance, and with or without an external oxidant at low temperatures. However, without photoredox catalysts, the earlier syntheses suffered from the dependence on external oxidants and harsh reaction conditions [83–84]. In this context, in 2014, Fabry et al. reported the use of novel dual photoredox catalytic systems made up of photoredox catalyst **11** and a rhodium catalyst to carry out the Weinreb amide C–H olefination shown in Scheme 1 [85]. By using this methodology, they assembled a library of compounds in good to excellent yields, with just 1 mol % of the photoredox catalyst **11** required. They observed that the yields of the products were dependent on various factors, such as the redox potential of the catalyst, the electronics of the ligand, and the nature of the reactive intermediates. Interestingly, without photoredox catalyst, no transformation was observed. In accordance with the plausible mechanism shown in Figure 6, chloride exchange between [RhCp*Cl_2_]_2_ and AgSbF_6_ generated the Rh(III) catalyst, which formed a five-membered rhodacycle **22** upon coordination with the carbonyl oxygen atom of the amide group. The complex **22** then coordinated to the acrylate product **23**, which rearranged to **24**, and the β-hydride elimination of **24** yielded the desired olefinated product **21**.

**Scheme 2:**
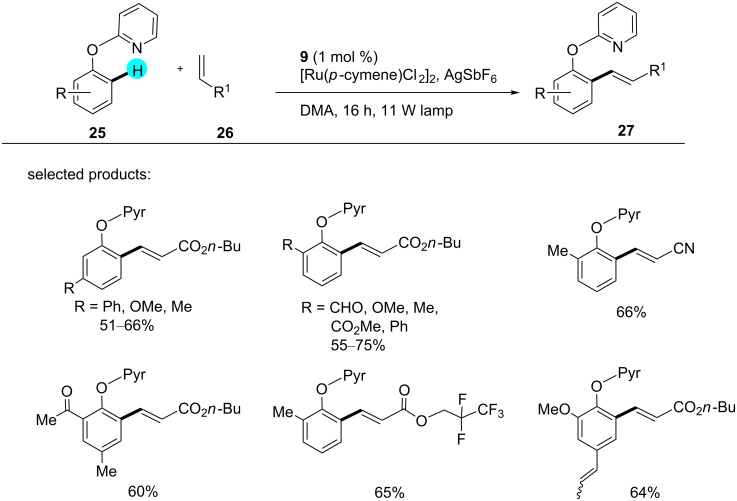
C–H olefination of phenolic ethers.

**C–H olefination of phenolic ethers:** Based on Ackermann and co-worker’s results [86], in 2015, Rueping and co-workers reported reoxidation reactions via visible photoredox catalysis [87]. In their study, they used photoredox catalyst **9** along with a Ru catalyst for *ortho* C–H functionalization of phenol derivatives, viz, *ortho*-(2-pyridyl)phenols (Pyr, Scheme 2). Captivatingly, in the absence of a photoredox catalyst, poor yields were obtained, and no reoxidation happened.

On the other hand, quite recently, the same group reported on the conversion of phenol derivatives using dual semiconductor photoredox catalysis for C–H bond functionalizations, which was proved to be a more sustainable procedure [88] in comparison to previously reported methods [89–92]. These semiconductor photocatalysts were better because they were: (i) cheap, (ii) easily separable from the reaction mixture, (iii) compatible with other transition metals, and (iv) provided steady reaction conditions. Similar to Scheme 2, there was no product obtained without a semiconductor photocatalyst. Therein, they utilized the efficient photoredox catalysts **16** (band gap: 2.4 eV) and **17** (band gap: 2.6–3.0 eV). However, better results were obtained with a heterogeneous semiconductor, photoredox catalyst **16**. In a semiconductor, the band gap is referred to as the difference in energy between the upper valence band and the lower conduction band. Upon irradiation, the electron holes in the band gap accept the electrons from the reduced moieties, thereby generating the C–H activation catalyst. In Figure 3, *E*_0,0_ is the energy gap between the ground state and the lowest triplet state, corresponding to the band gap in semiconductors.

#### C–H acylation

**Scheme 3:**
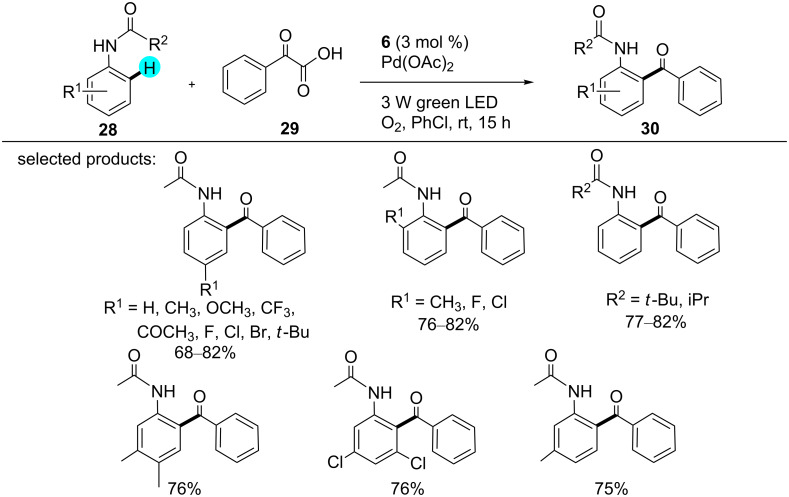
Decarboxylative acylation of acetanilides.

**Figure 7:**
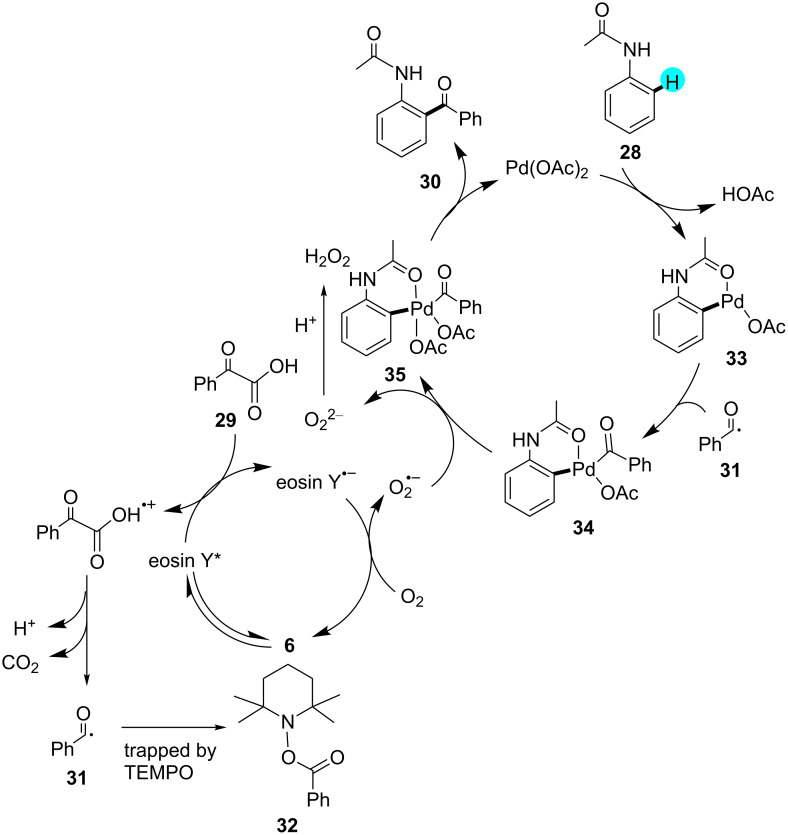
Mechanism for the formation of **30** from acetanilide derivatives.

**Decarboxylative acylation of acetanilides:** In 2015, Wang and co-workers first reported the acylation of acetanilides via C–H functionalization using photoredox catalyst **6** [93]. Significantly, the use of this organic dye was much more feasible and economical as compared to other transition metal photoredox catalysts. The group worked with a dual visible light photoredox catalytic system by combining photoredox catalyst **6** with a Pd catalyst in the presence of molecular oxygen as an oxidant. Although various photoredox catalysts and solvents were examined, the best results were obtained with photoredox catalyst **6** in chlorinated solvents. In the absence of a photoredox catalyst, the goup did not observe any product formation. A list of products assembled through this methodology is shown in Scheme 3, and the mechanistic pathway that is involved is displayed in Figure 7.

**Scheme 4:**
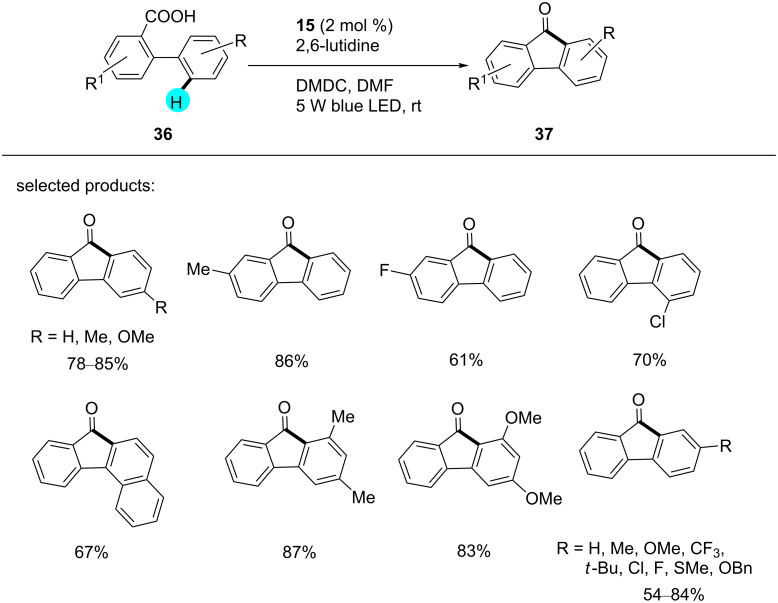
Synthesis of fluorenone derivatives by intramolecular deoxygenative acylation of biaryl carboxylic acids.

**Figure 8:**
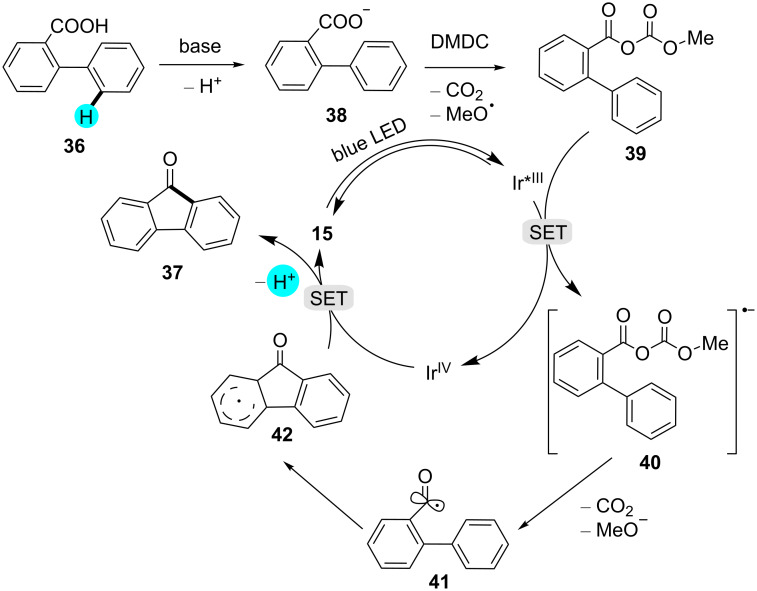
Mechanism for the photoredox-catalyzed synthesis of fluorenone derivatives.

**Synthesis of fluorenones:** Very recently, Ruzi et al. generated several fluorenone derivatives via dual photoredox-catalyzed deoxygenative intramolecular acylation reactions at room temperature (Scheme 4) [94]. In their study, they observed that electron-donating groups provided better yields as compared to electron-withdrawing groups. The earlier reported methods for the synthesis of fluorenones, viz, Friedel–Craft acylations [95–96], oxidations of fluorenes [97–98], and Diels–Alder reactions [99], generated a lot of waste products. Therefore, this methodology seemed to be far better as compared to previously reported methods. With the help of this strategy, the group assembled several fluorenones that could further be functionalized to generate other interesting molecules. As can be seen in Figure 8, the mechanism of the reaction commences with the deprotonation of the biphenyl carboxylic acid **36**, followed by the reaction of **38** with dimethyl dicarbonate (DMDC) to generate compound **39**. On the other hand, the photocatalyst is excited by metal–ligand charge transfer, which produces an intermediate radical anion **40** via SET. Then, the intermediate **40** yields the acylated radical **41** by fragmentation, which, upon intramolecular addition, followed by one-electron oxidation and deprotonation, gives the desired product **37**.

#### C–H thiolation

**Scheme 5:**
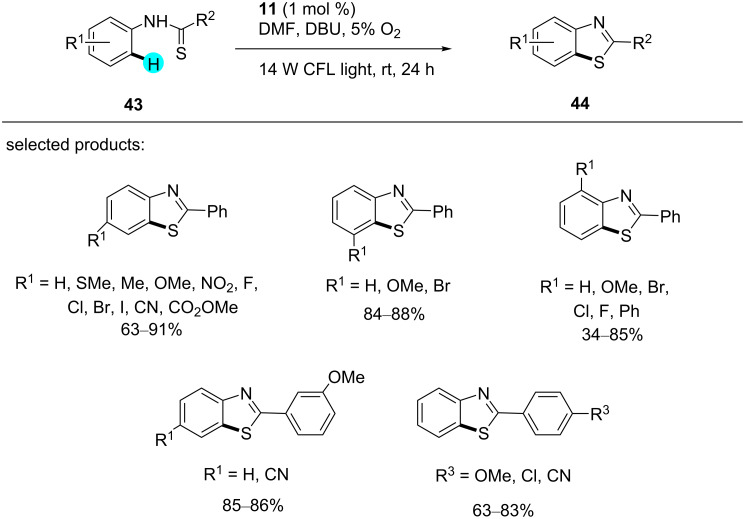
Synthesis of benzothiazoles via aerobic C–H thiolation.

**Figure 9:**
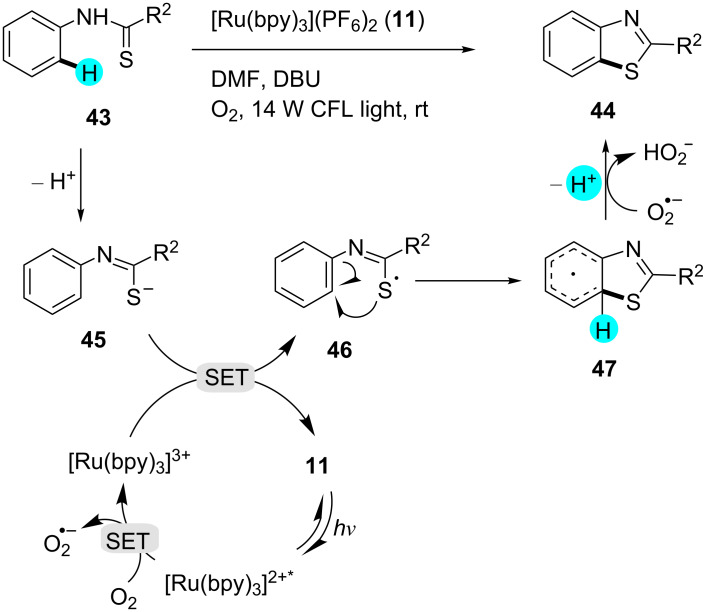
Plausible mechanism for the construction of benzothiazoles from benzothioamides.

**Synthesis of benzothiazoles via aerobic C–H thiolation:** In 2011, Cheng et al. reported a mild and efficient synthetic process for the construction of benzothiazoles, which have many applications in biology and pharmacy, via C–H functionalization without the direct involvement of a metal, using visible light-mediated photoredox catalysis [100]. They observed a number of interesting facts, viz: (i) the reaction proceeded with environmentally friendly molecular oxygen as oxidant, (ii) water was the only byproduct of the reaction, (iii) no reaction occurred without the involvement of a photocatalyst, (iv) high yields were obtained with electron-donating substituents (Scheme 5), and (v) the rate-determining step (i.e., breaking of the C–H bond) was suggested to follow a first-order kinetic isotope effect (*K*_H_/*K*_D_ = 5). As such, a library of benzothiazole derivatives was reported using this methodology, and a plausible mechanism is shown in Figure 9.

**Scheme 6:**
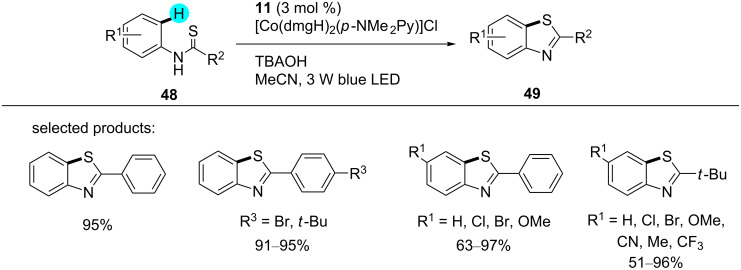
Synthesis of benzothiazoles via oxidant-free C–H thiolation.

**Figure 10:**
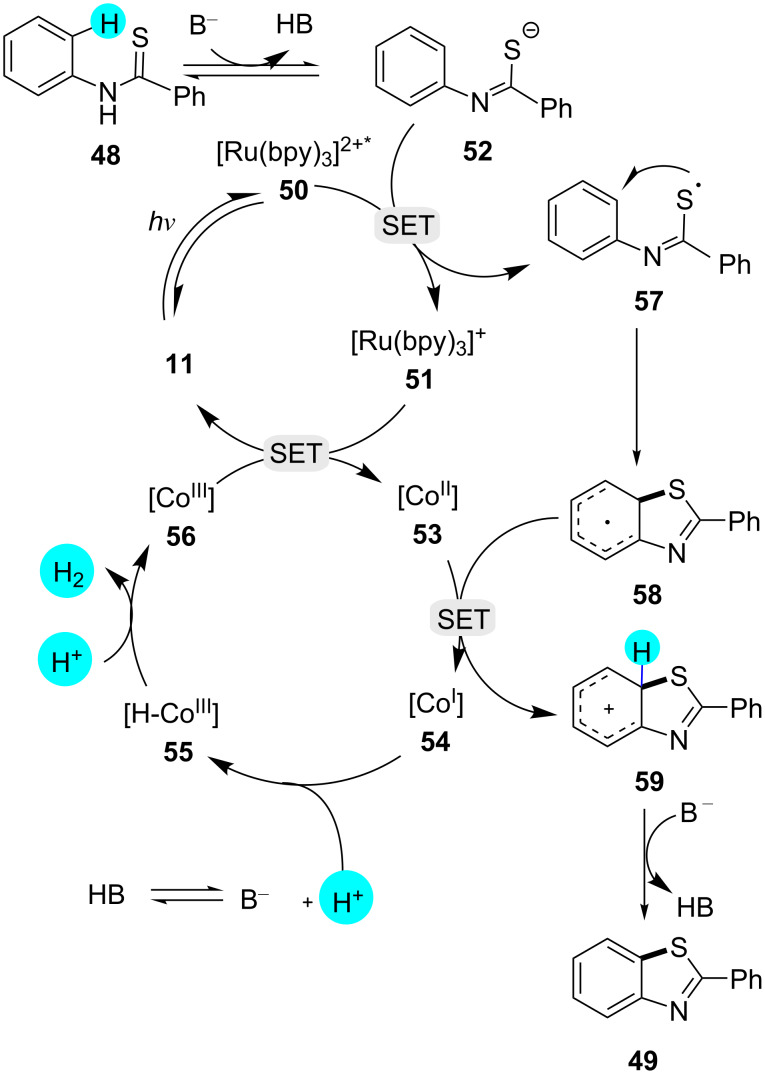
Mechanism involved in the synthesis of benzothiazoles via oxidant-free C–H thiolation.

**Synthesis of benzothiazoles via oxidant-free C–H thiolation:** On the other hand, a novel dual photoredox catalytic system of photoredox catalyst **11** and a cobalt catalyst was designed by Wu and Lei for the construction of benzothiazoles [101]. Because of the properties of the photoredox catalyst **11**, the reaction was carried out without the requirement of a proton acceptor or external oxidant, and hydrogen gas was obtained as the only side product (Scheme 6). This was a major achievement since the previously reported methods suffered from undesirable side products and the requirement of oxidants [102–103]. After the use of various bases, however, the best results were obtained with tetrabutylammonium hydroxide (TBAOH). The suggested mechanism commences with the photoexcitation of photocatalyst **11** to a strongly oxidizing excited state **50** (*E*_1/2 red_([Ru(bpy)_3_]^2+*/^[Ru(bpy)]^3+)^ = +0.77 V vs SCE). Then, SET from anion **52** takes place for the generation of the S-centered radical **57**. The reactive aryl radical **58** is obtained by the cyclization of the sulfur-based radical **57**. Simultaneously, photocatalyst **11** is regenerated from Ru(I) photocatalyst **51** and Co(III) complex **56**. Additionally, the Co(I) complex **54** and the cation **59** are obtained by the reduction of the Co(II) catalyst **53** by radical **58**. In order to form the desired cyclized product, rearomatization of **59** takes place, and protonation of **55** assists the regeneration of **56**, with parallel hydrogen release, as shown in Figure 10.

#### C–H cyclization

**Scheme 7:**
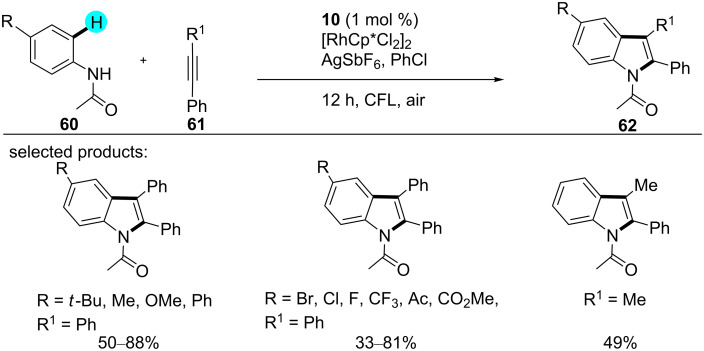
Synthesis of indoles via C–H cyclization of anilides with alkynes.

**Synthesis of coumarins and indoles:** Coumarins and indoles are a large class of compounds gifted with a rich and attractive chemistry. They are found in numerous bioactive natural and nonnatural products. Therefore, a lot of work has been devoted to the construction of diverse intricate molecules containing these scaffolds in their structures [104–107]. By employing dual photoredox catalysis, in 2016, Fabry et al. reported the cyclization of substituted anilides with alkynes to produce indoles [108]. Unlike previously reported syntheses, viz, an indole synthesis by the Fagnou group utilizing a large amount of copper as an oxidant [109–110], this reaction was carried out under mild reaction conditions in the presence of photoredox catalyst **10** and a Rh catalyst (Scheme 7). They also reported another Pd-catalyzed indole synthesis with photoredox catalyst **9**, similar to the Rh-catalyzed synthesis [108]. From mechanistic studies it can be inferred that the photoredox catalysis process is independent from the C–H activation process. They also successfully demonstrated the importance of a photoredox catalysts in the generation of superoxide radicals.

**Scheme 8:**
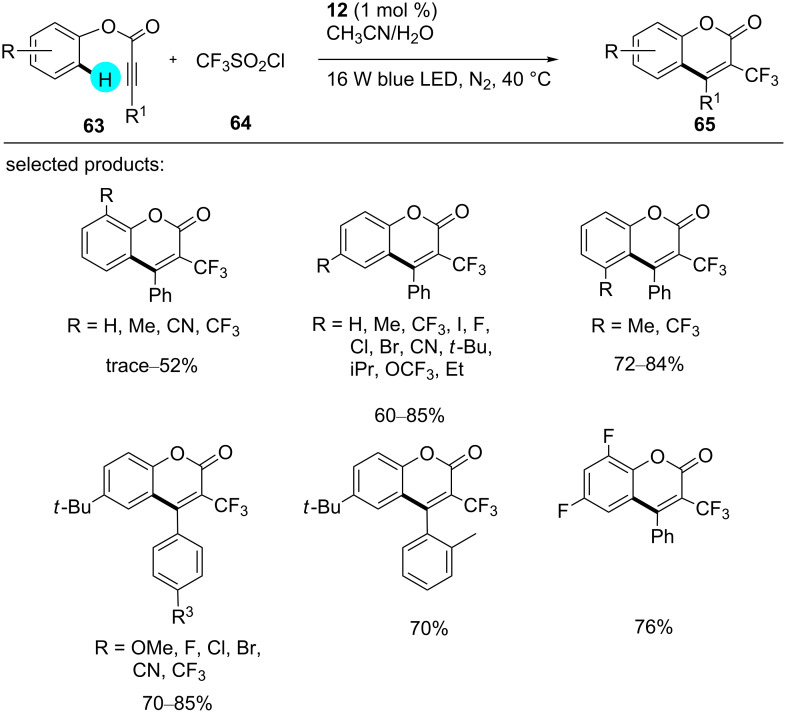
Preparation of 3-trifluoromethylcoumarins via C–H cyclization of arylpropiolate esters.

**Figure 11:**
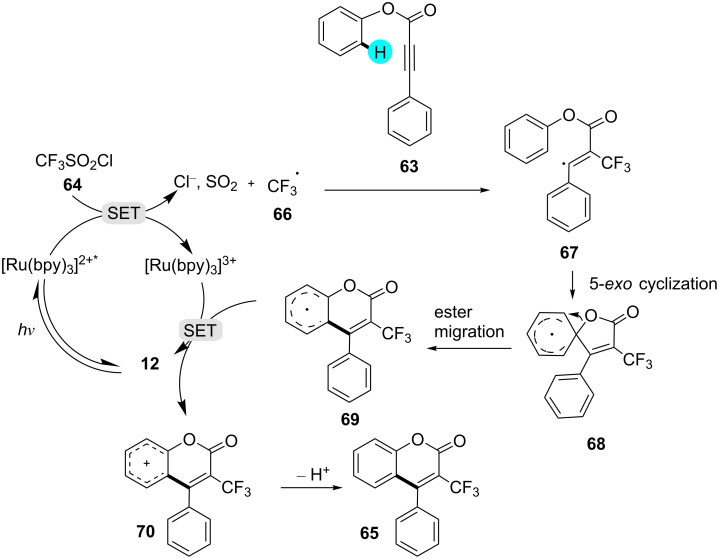
Mechanistic pathway for the synthesis of coumarin derivatives via C–H cyclization.

On the other hand, recently, Xiong and co-workers reported the synthesis of coumarin derivatives using photoredox catalyst **12** and CF_3_SO_2_Cl as a potent radical source [111]. In comparison to other reported methods, the reaction was carried out under mild as well as environmentally friendly conditions, and the reaction remarkably showed much tolerance for various functional groups (Scheme 8). The detailed proposed mechanism for the constructions of these intriguing molecules is shown in Figure 11.

### C–H benzoyloxylation: monobenzoyloxylation without chelation assistance

**Scheme 9:**
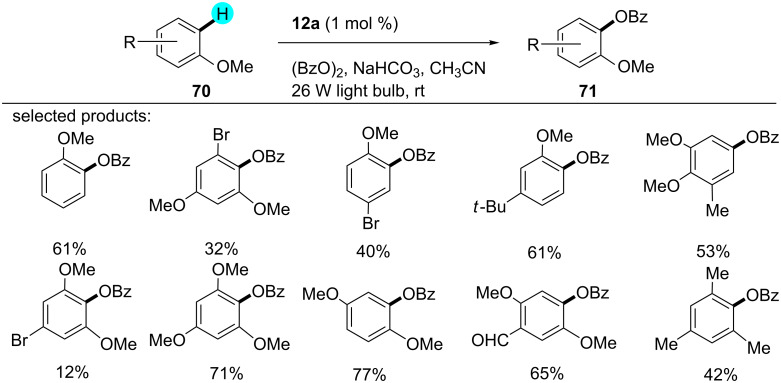
Monobenzoyloxylation without chelation assistance.

**Figure 12:**
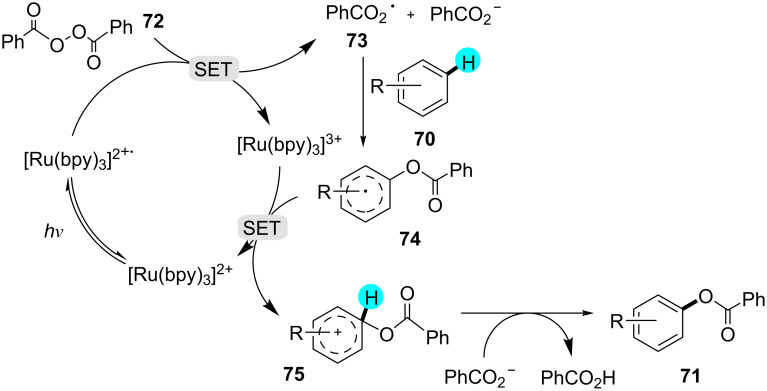
Plausible mechanism for the formation of **71** from **70**.

Inspired by the innovative work reported by Tokumaru [112] and MacMillan [113], Li’s group reported a visible light-induced aryl C–H monobenzoyloxylation with photoredox catalyst **12a** (Scheme 9) [114]. The *ortho*- and *para*-substituted products were obtained in a 1.9:1 ratio, with no decarboxylated byproducts. The proposed mechanism proceeds with the excitation of the photocatalyst, followed by the reduction of **72** to PhCO_2_^−^ along with the generation of the radical **73**, which further attacks the electron-richest position of **70**. Next, the reactive cation species **75** is generated via an SET mechanism. In the end, PhCO_2_^−^ abstracts a proton, which yields the benzoyloxylated product **71**, as shown in Figure 12. Likewise, no result was obtained in the absence of light and photocatalyst.

#### Aryl C–H arylation

**Scheme 10:**
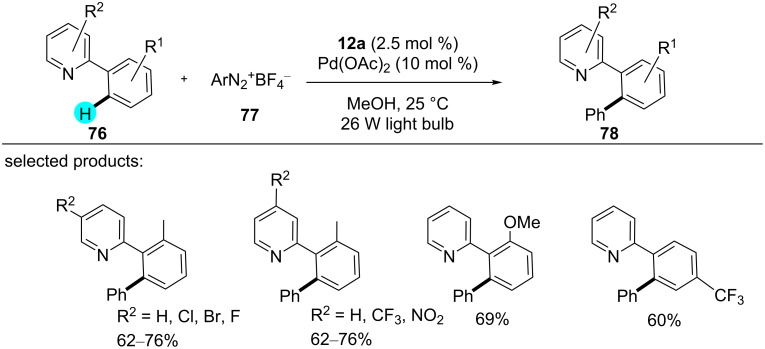
Aryl-substituted arenes prepared by inorganic photoredox catalysis using **12a**.

**Figure 13:**
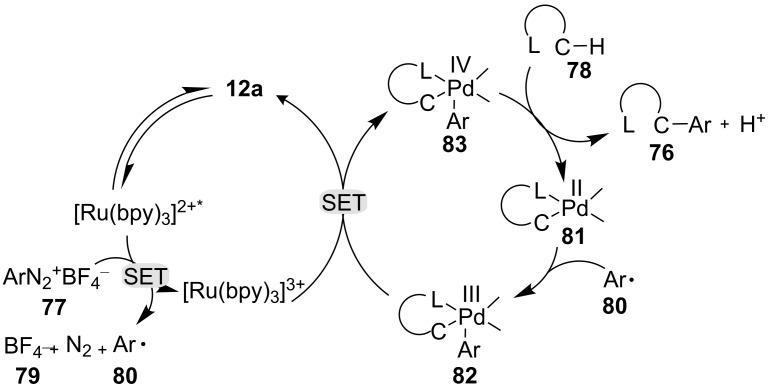
Proposed mechanism for C–H arylations in the presence of **12a** and a Pd catalyst.

With the idea of introducing dual photoredox catalysis, in 2011, Sanford’s group reported the preparation of arylated compounds [115], important structural components of many natural products, organic materials, etc., in such a way [116–117]. They used a Pd catalyst and a photoredox catalyst **12a** at room temperature to generate reactive intermediates (Scheme 10) [115]. This novel method was applicable to various directing groups and had a high functional group tolerance, whereas the previously reported methods required high temperatures [118–120]. A library of compounds was reported by that group using this approach, and a plausible mechanism is shown in Figure 13.

**Scheme 11:**
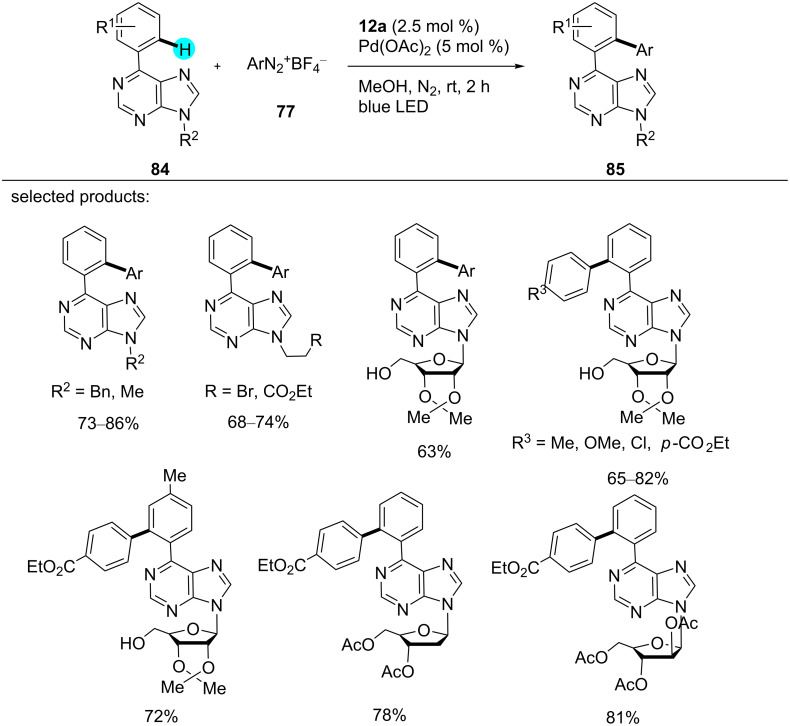
Arylation of purines via dual photoredox catalysis.

**Arylation of purines:** Purine bases and purine nucleosides, which are common structural motifs in DNA and RNA, have an enormous range of applications in biology [121–123]. Inspired by other C–H arylation methods for N-heterocycles [124–126], recently, Guo and co-workers reported a dual photoredox-catalyzed C–H arylation of 6-arylpurine using photoredox catalyst **12a** in the presence of a Pd cocatalyst [127]. With the aid of photoredox catalyst **12a**, the reaction took place under mild conditions with high regioselectivity and excellent functional group tolerance, as shown in Scheme 11 [127].

**Scheme 12:**
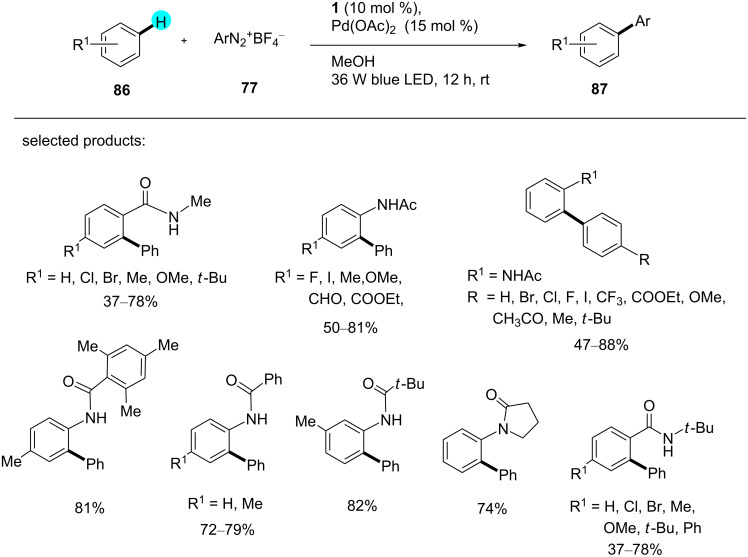
Arylation of substituted arenes with an organic photoredox catalyst.

On the other hand, Xu and co-workers reported the preparation of arylated products via dual photoredox catalysis using the competent organic photoredox catalyst 9,10-dihydroacridine (**1**) under mild conditions at room temperature [128]. As usual, no reaction was observed without photoredox catalyst **1** or LED light. Mechanistic studies showed that the reaction proceeded via the generation of an aryl radical. As such, a list of products was assembled with moderate to high yields using this powerful synthetic strategy, and the scope of the reaction can be seen in Scheme 12.

#### C–H trifluoromethylation

**Scheme 13:**
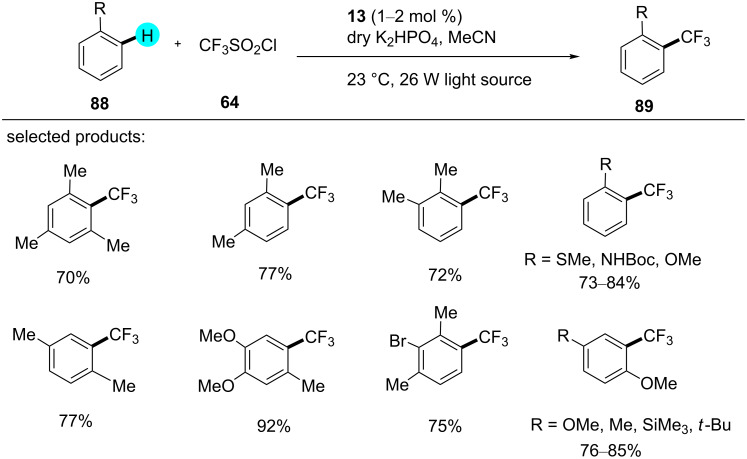
C–H trifluoromethylation.

**Figure 14:**
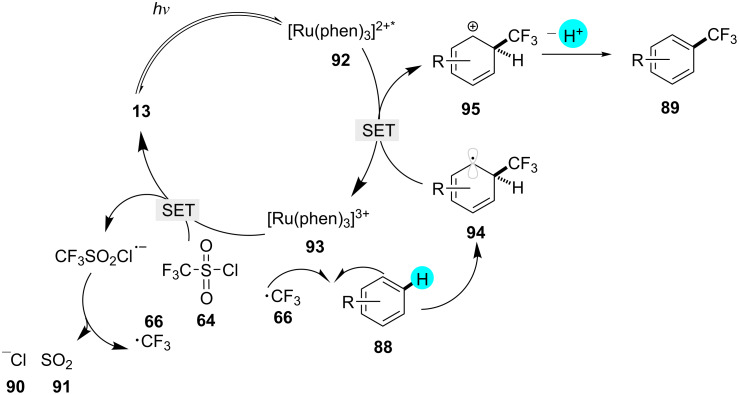
Proposed mechanism for the trifluoromethylation of **88**.

The introduction of a CF_3_ group into pharmaceutical agents can enhance their performance in medicinal chemistry [129–131]. In 2011, Nagib and MacMillan reported the synthesis of a broad scope of trifluoromethylated products via a radical-mediated methodology using photoredox catalyst **13** (Scheme 13) [113]. In comparison to other reported methods that used harsher reaction conditions, they carried out the reaction using trifluoromethanesulfonyl chloride, which is easier to handle and cost-efficient. By using this methodology, they prepared diverse trifluoromethylated derivatives, which have applications in medicinal chemistry. The mechanism of the reaction involves the excitation of the photocatalyst **13**, generating **92**. The reduction of triflyl chloride (**64**) by SET gives the highly energetic compound **66**, which combines with **88** to give **94**. The oxidation of **94** by **92** generates an intermediate **95**, which, upon further deprotonation, produces the desired product **89** (Figure 14).

#### C–H lactonization: synthesis of benzo-3,4-coumarins

**Scheme 14:**
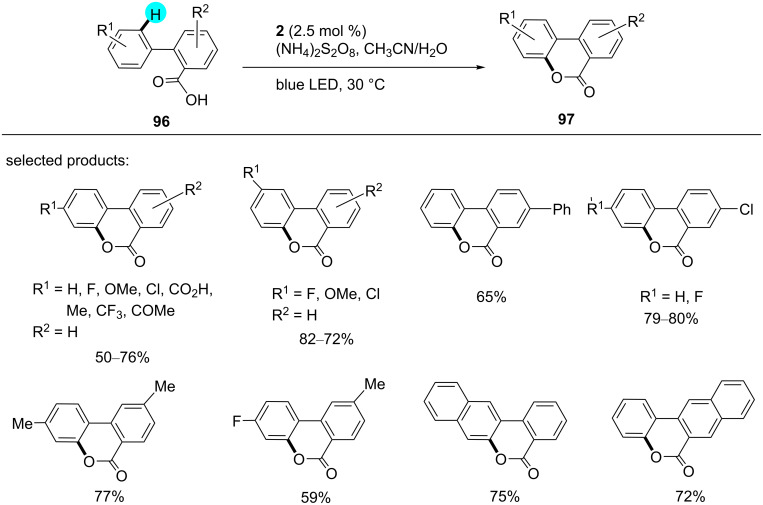
Synthesis of benzo-3,4-coumarin derivatives.

**Figure 15:**
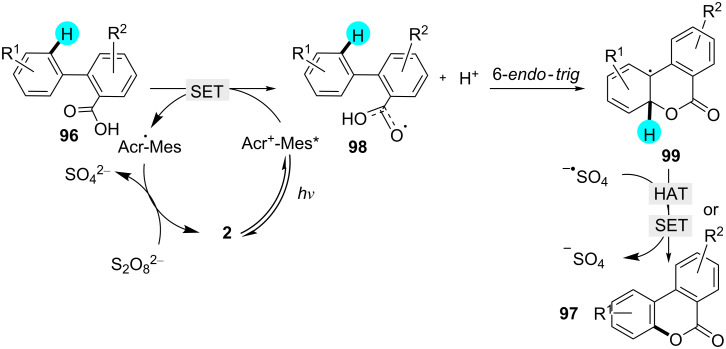
Plausible mechanism for the synthesis of substituted coumarins.

Benzo-3,4-coumarins are a key intermediate for diverse natural products, and they have already been synthesized by various research groups with different procedures [132–138]. Inspired by previous work, Gomez and co-workers reported the preparation of benzo-3,4-coumarins using photoredox catalyst **2** with (NH_4_)_2_S_2_O_8_, which acted as a cost-efficient and environmentally friendly oxidant (Scheme 14) [139]. In the mechanism, the excited photocatalyst generates the benzoyloxy radical **98**, and the cyclization of **98** is completed via 6-*endo*-*trig* ring formation to form the intermediate **99**, which, upon oxidation via HAT or SET/deprotonation, generates the desired product **97** (Figure 15). The oxidation of the persulfate anion generates a sulfate radical anion, which acts as an oxidant in the aromatization step. In the absence of light and photoredox catalyst, no product was obtained.

#### C–H phosphonylation: oxidant-free oxidative phosphonylation

**Scheme 15:**
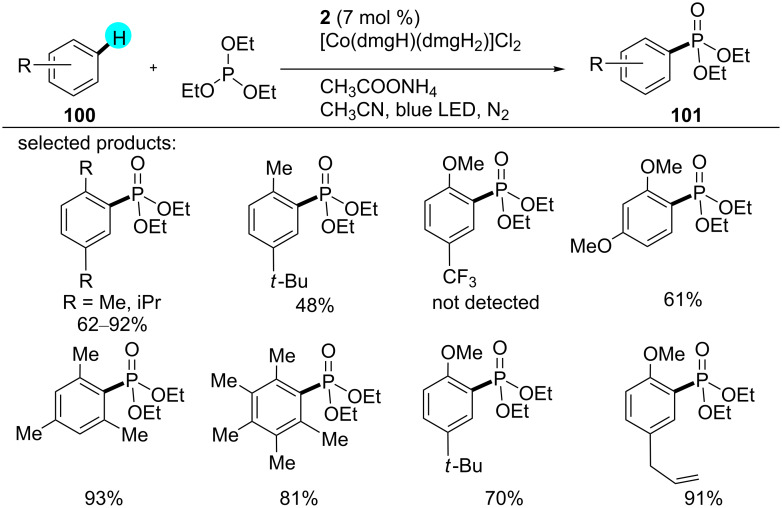
Oxidant-free oxidative phosphonylation.

**Figure 16:**
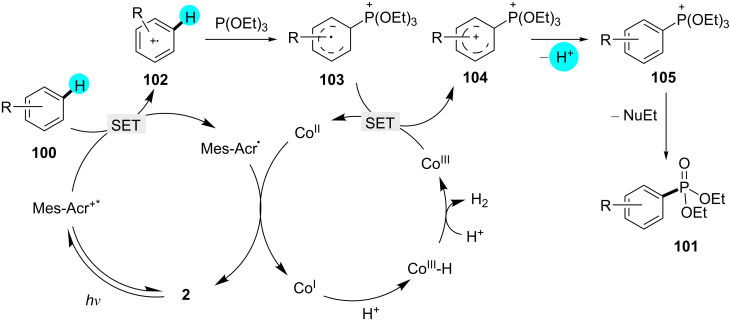
Mechanism proposed for the phosphonylation reaction of **100**.

Considering the importance of phosphonylation, recently, Lei and co-workers reported a novel method for the synthesis of phosphonylated products using photoredox catalyst **2** along the cocatalyst [Co(dmgH)(dmgH_2_)]Cl_2_ [140]. Although earlier approaches showed good regioselectivity, the requirement of directing groups and preactivation of the compounds were the major drawbacks [141–143]. On the other hand, when the reaction was carried out with photoredox catalyst **6** and **11**, respectively, no product was obtained, showcasing the necessity of a highly oxidizing photoredox catalyst for the oxidation of the arenes. The reaction is initiated by the oxidation of **100** through the excited photocatalyst to generate the arene radical cation **102**. Here, P(OEt)_3_ acts as a nucleophile, capturing the radical cation of **102** and generating **103**. Concomitant to the reduction of the Co(III) catalyst to Co(II), the arene intermediate **104** is generated via SET, and after deprotonation of **104**, the phosphonylated intermediate **105** is formed. The additive CH_3_COONH_4_ causes nucleophile displacement, converting the arylphosphonium salt into the phosphonylated product **101**. A range of products was assembled using this strategy, as displayed in Scheme 15, and a plausible mechanism for the reaction is shown in Figure 16.

#### C–H nitration of protected anilines

**Scheme 16:**
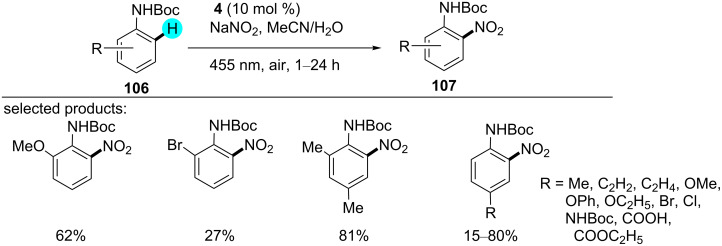
Nitration of anilines.

**Figure 17:**
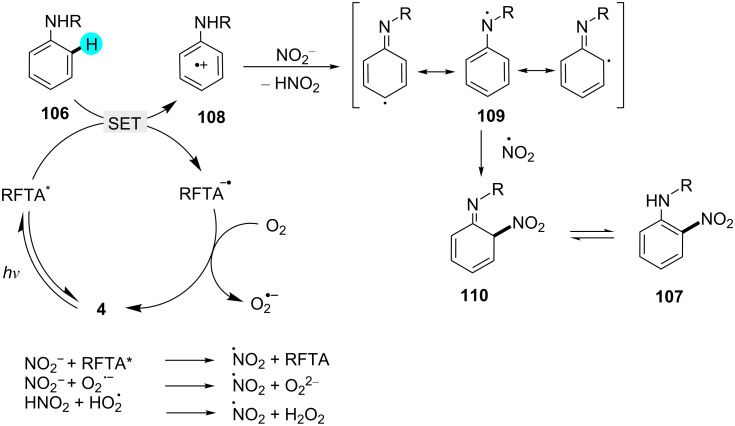
Plausible mechanism for the nitration of aniline derivatives via photoredox catalysis.

Nitroanilines are an important class of compounds and found in many drugs and dyes [144]. Therefore, recently, König and co-worker reported the synthesis of protected anilines by employing organic photoredox catalyst **4** with sodium nitrite as a cost-efficient source of NO_2_ at room temperature (Scheme 16) [145]. They also tried to perform the same reaction in the absence of light and photocatalyst, but no reaction progress was observed. As reported in the literature, earlier methods applied harsher conditions, such as a strong acid and high temperature. Although in some cases, a mild nitration agent such as *tert*-butyl nitrite (TBN) was used, the reaction required an elevated temperature [146–150]. As can be seen in Figure 17, the reaction is initiate with the excitation of the photocatalyst, which further oxidizes the aniline derivative **106** to generate the arene radical cation **108**. Then, the intermediate **109** is formed by deprotonation, which, upon reaction with a nitrate radical, gives the desired product **107**.

#### Aryl C–H amination of *ortho* positions: synthesis of carbazoles

**Scheme 17:**
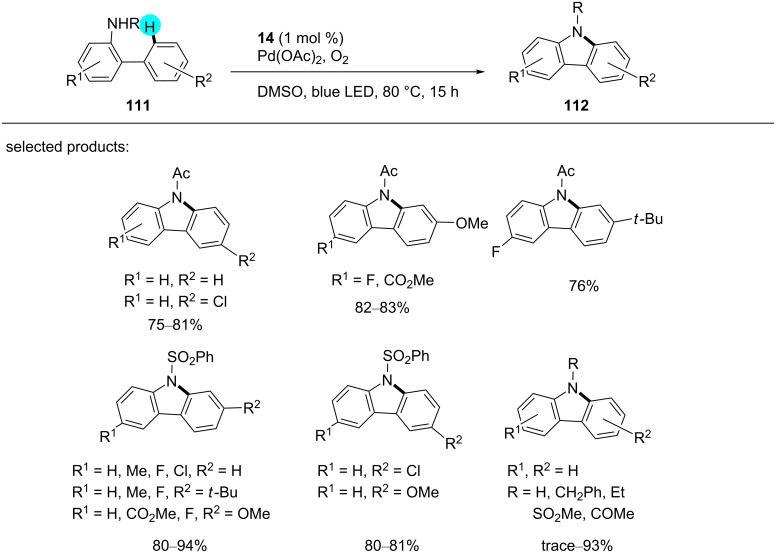
Synthesis of carbazoles via intramolecular amination.

**Figure 18:**
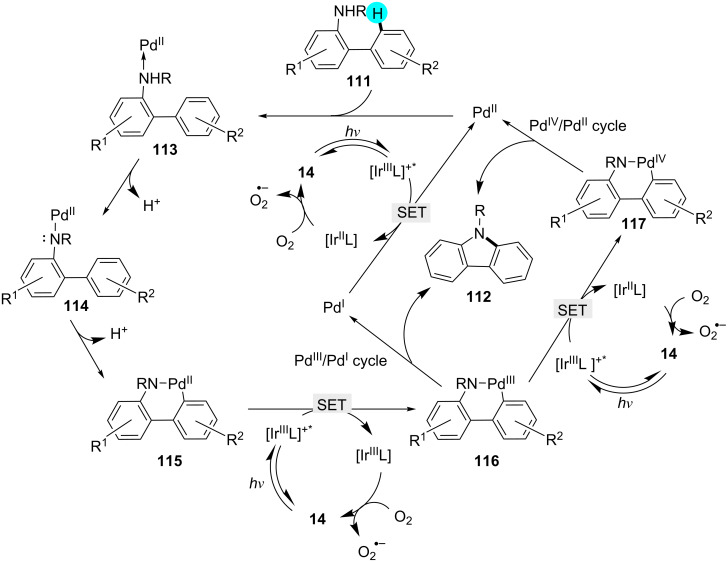
Proposed mechanism for the formation of carbazoles from biaryl derivatives.

Although a handful of methods for the construction of carbazoles, which are biologically important, is available in the literature, these procedures suffer from the need for an elevated temperature and the requirement of stoichiometric amounts of strong oxidants [151–153]. To overcome these drawbacks, recently, Cho’s group synthesized carbazole derivatives using a dual photoredox-catalyzed intramolecular C−H bond amination of *N*-substituted 2-amidobiaryls with photoredox catalyst **14** in presence of Pd(OAc)_2_ as a cocatalyst under aerobic conditions [154]. The substrate scope is displayed in Scheme 17, and the proposed mechanism is shown in Figure 18.

### Aryl *para* C–H bond functionalization

Taking forward the prevalent research on direct C–H bond functionalization strategies, the substitution of *para* C–H bonds through the application of photoredox catalysts was also reported, and this approach allowed for easy and fast transformations to take place. Herein, we cover all the reported strategies for aryl *para* C–H bond functionalizations by means of photoredox catalysis.

#### Aryl C–H hydroxylation: synthesis of substituted phenols

**Scheme 18:**
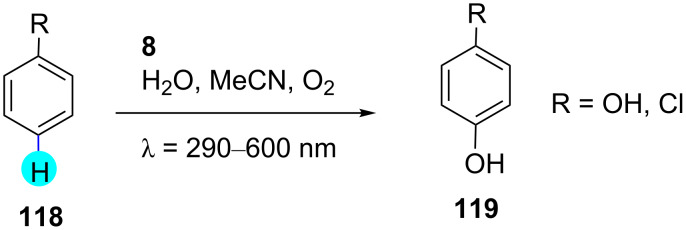
Synthesis of substituted phenols using QuCN.

**Figure 19:**
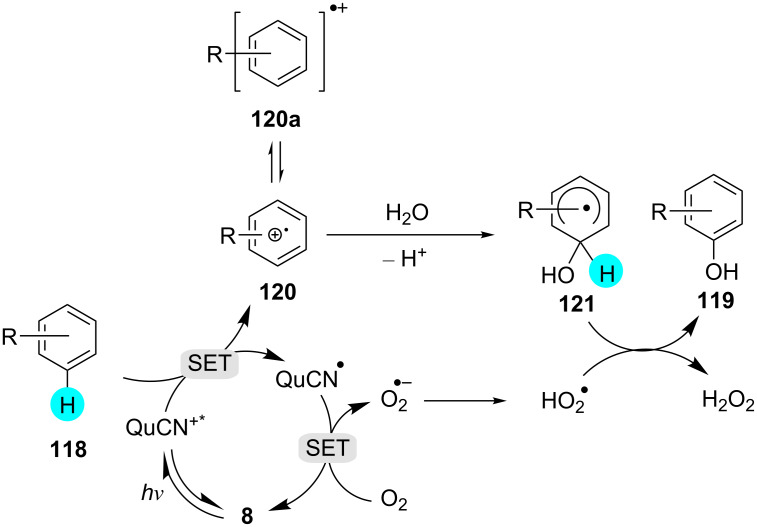
Mechanism for the synthesis of phenol derivatives with photoredox catalyst **8**.

The synthesis of phenol derivatives using the cumene process or single-step oxygenation suffers from poor yields, high reaction temperatures, and high-energy UV irradiation conditions [155–156]. To overcome this, in 2012, Fukuzumi and co-workers used 3-cyano-1-methylquinolinium perchlorate (**8**) as a photoredox catalyst for the hydroxylation of arenes (Scheme 18) [157]. They realized that the photoredox catalyst **8** possesses a great oxidizing ability (*E*_red_ vs SCE = 2.72 V) at ambient conditions. The mechanism of the reaction was studied by fluorescence quenching and transient absorption spectroscopy. They observed that the one-electron reduction potential of ^1^QuCN^+*^ was higher than that of benzene (*E*_ox_ vs SCE = 2.32 V), making the electron transfer from phenol to ^1^QuCN^+^* viable. The mechanistic pathway for the C–H hydroxylation of benzene derivatives is shown in Figure 19.

**Scheme 19:**
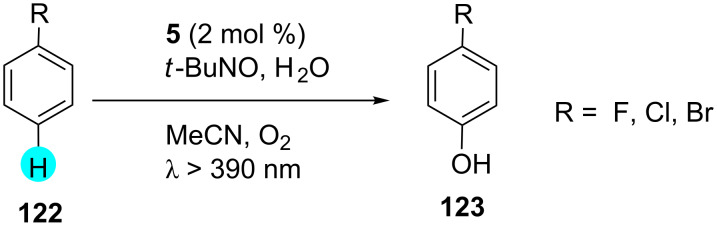
Synthesis of substituted phenols with DDQ (**5**).

**Figure 20:**
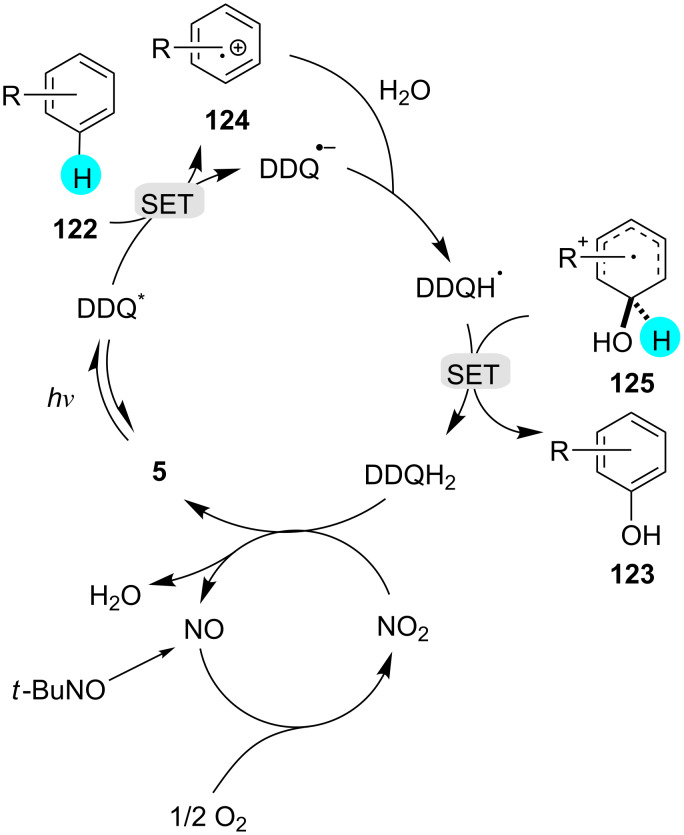
Possible mechanism for the generation of phenols with the aid of photoredox catalyst **5**.

On the other hand, Ohkubo et al. reported that substrates like benzene are difficult to oxidize with nonphotochemical processes, whereas with photoredox catalyst **5** and TBN in the presence of oxygen, benzene can be easily be oxidized (Scheme 19) [158]. Remarkably, photoredox catalyst **5** offered a one-step oxygenation of arenes to phenol with high quantum yields. It was observed that hydroxylation of the fluoro-, chloro-, and bromobenzene derivatives provided low yields. The photocatalytic mechanism for this reaction was inspected by time-resolved transient absorption spectroscopy to detect the triplet–triplet photoredox catalyst spectrum via nanosecond laser flash photolysis. The mechanism involved in this transformation is shown in Figure 20.

#### Aryl C–H halogenation

**Scheme 20:**
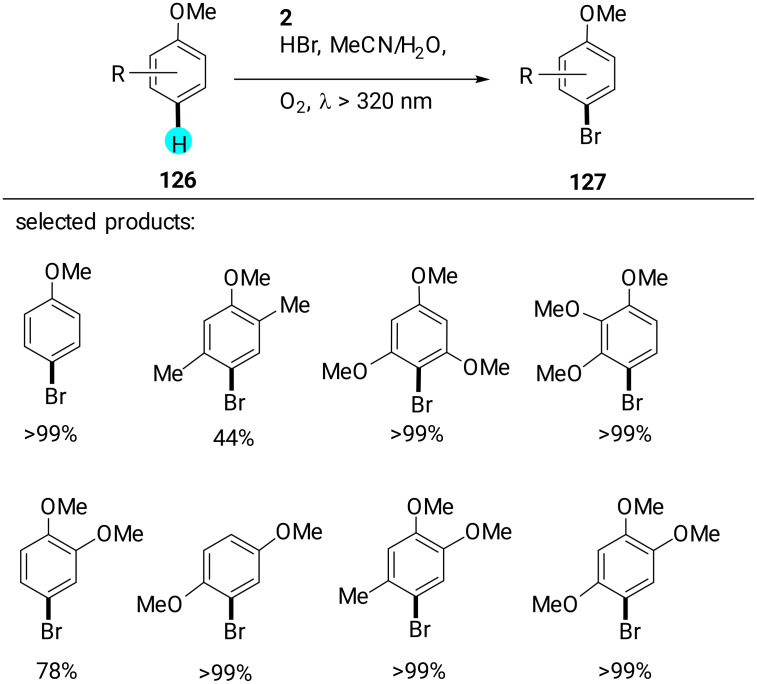
Aerobic bromination of arenes using an acridinium-based photocatalyst.

**Aerobic bromination of arenes:** In another experiment, Ohkubo et al. reported that for aerobic aryl C–H brominations, HBr can be utilized with photoredox catalyst **2** in the presence of molecular oxygen to yield the monobrominated products in excellent yields via visible-light photoredox catalysis (Scheme 20) [159]. Earlier literature reports suffered from the use of hazardous compounds (e.g., bromine) and low selectivities [160]. The reactive radical intermediates in their study were detected via laser flash photolysis measurements, and the monobrominated product selectivity was controlled by the difference in electron transfer oxidation reactivity of **127** and **126** of electron transfer state of photocatalyst (Me**^·^**^+^ moiety) and also on the radical cations reactivity with Br^−^.

**Scheme 21:**
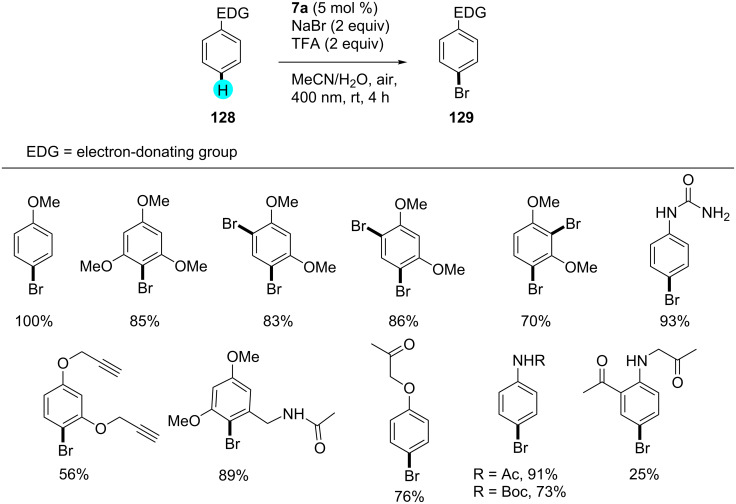
Aerobic bromination of arenes with anthraquinone.

**Figure 21:**
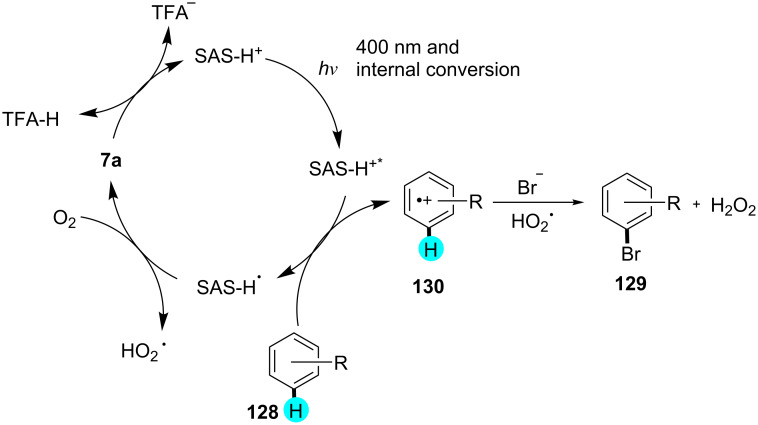
Proposed mechanism for the synthesis of monobrominated compounds.

Recently, König’s group reported on photocatalytic brominations using a stronger oxidizing photocatalyst, viz, sodium anthraquinone-2-sulfonate (SAS, **7a**, 2.3 V vs SCE) [161–162]. In their studies, they did not only observe excellent regioselectivities but also great functional group tolerance under mild reaction conditions. For efficient brominations, they employed sodium bromide in the presence of oxygen. The activation of the photocatalyst through protonation was shown by cyclic voltammetry, and the other interactions were revealed by emission quenching experiments and UV–vis spectroscopy. As can be seen in Scheme 21, the group prepared a library of monobrominated compounds using this simple yet effective strategy. A plausible mechanism is shown in Figure 21.

**Scheme 22:**
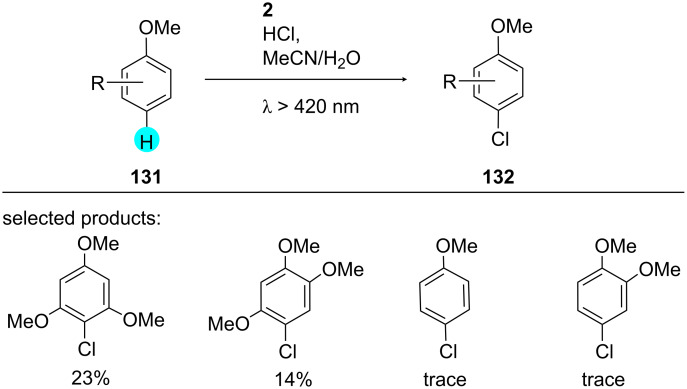
Chlorination of benzene derivatives with Mes-Acr-MeClO_4_ (**2**).

**Figure 22:**
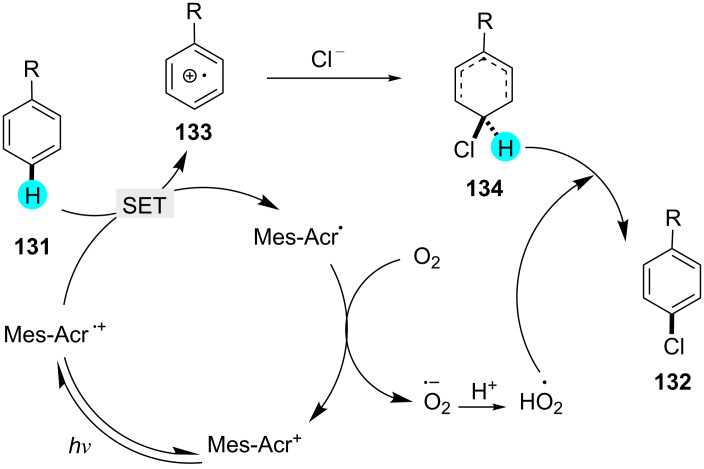
Mechanism for the synthesis of **131** from **132**.

**Chlorination of arenes with Mes-Acr-MeClO_4_ (2):** Ohkubo et al. observed that only under aerobic photocatalytic conditions, C–H chlorination of trimethoxybenzene (TMB) occurs [163]. They exploited the potent photoredox catalyst **2** for the excitation of the substrate. The mechanism was detected by nanosecond transient absorption spectroscopy. The substrate scope and mechanism are shown in Scheme 22 and Figure 22.

**Scheme 23:**
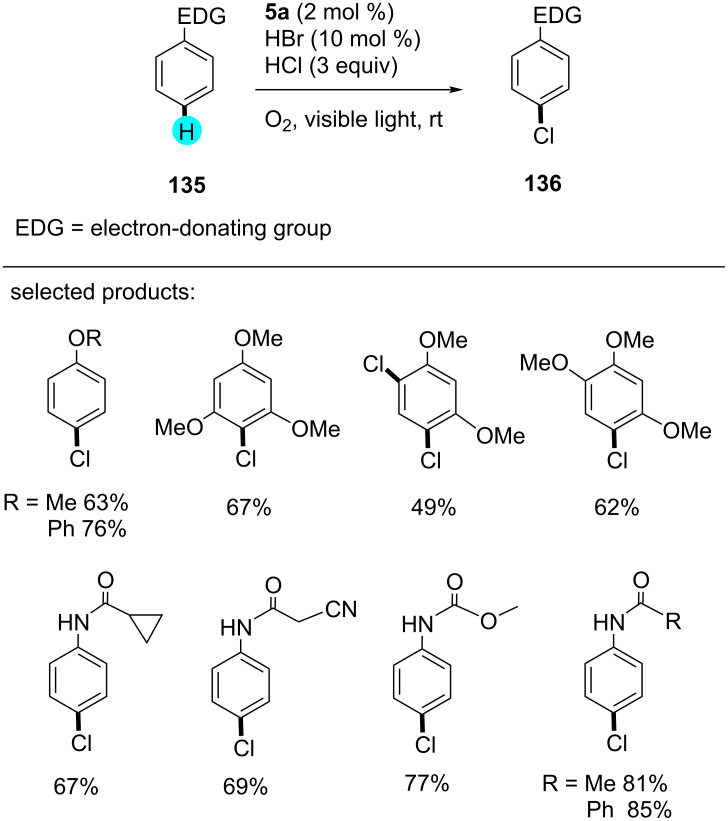
Chlorination of arenes with 4CzIPN (**5a**).

**Figure 23:**
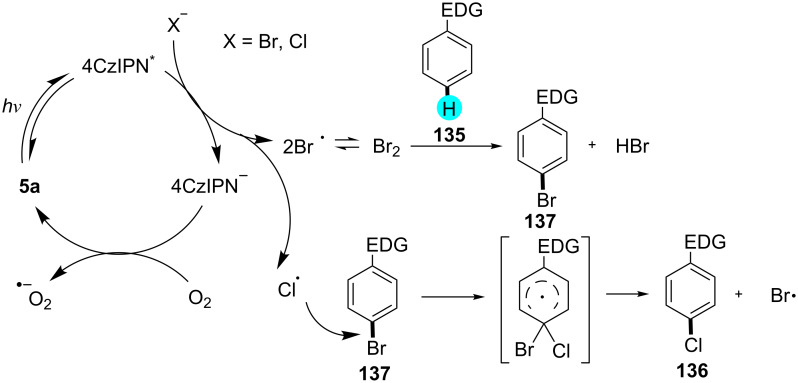
Plausible mechanism for the oxidative photocatalytic monochlorination using **5a**.

Furthermore, König and co-workers synthesized monochlorinated compounds via an oxidative photocatalytic pathway [164]. They performed the reaction in the presence of organic photoredox catalyst 1,2,3,5-tetrakis(carbazol-9-yl)-4,6-dicyanobenzene (**5a**, 4CzIPN), environment friendly oxygen, and hydrochloride as a chloride source. The activation proceeded via bromination in situ, followed by *ipso*-chlorination, which yielded the desired products with high regioselectivity. The substrate scope is displayed in Scheme 23, and the mechanism involved in this transformation is shown in Figure 23.

**Scheme 24:**
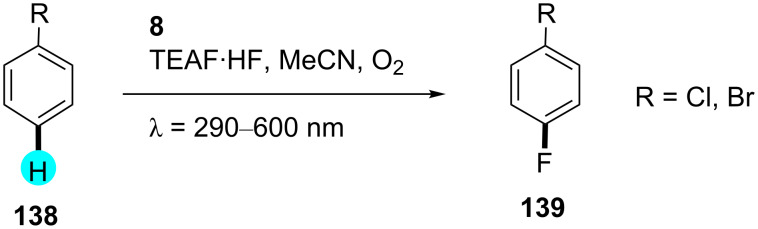
Monofluorination using QuCN-ClO_4_ (**8**).

**Monofluorination of arenes:** Direct monofluorination has always been a challenging task in synthetic organic chemistry. Although several methods are available for direct fluorine introduction in the literature, most of them suffer from poor yields and low selectivities [165–167]. In this context, Fukuzumi and co-workers reported the transformation of C–H bonds into C–F bonds in the presence of photoredox catalyst **8**, with a similar mechanism as shown in Figure 22, and the photocatalytic mechanism was elucidated by nanosecond laser flash photolysis (Scheme 24) [168].

**Scheme 25:**
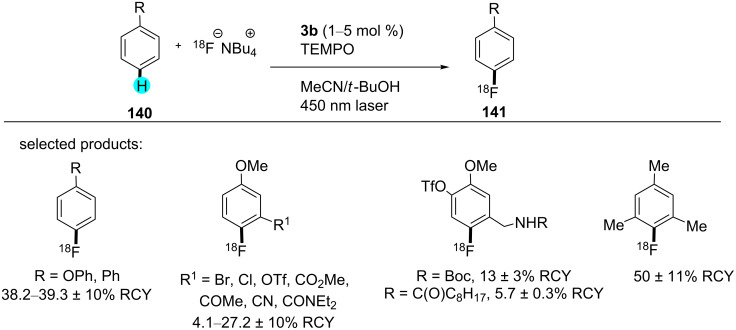
Fluorination with fluorine-18.

Fluorine-18 is an important radioisotope used in the radiopharmaceutical industry (e.g., for drug delivery, medical imaging, etc.). Therefore, functionalization of arenes with this isotope is an important task. Although several reports are available in the literature for these transformations, they suffer from poor reactivity, unreactive starting material, and lower activities of the ^18^F-labeled tracers. In this context, Li’s group has recently developed an effective and mild technique for C–H ^18^F fluorination with the aid of visible-light photoredox catalysis (Scheme 25) [169]. Using this approach, several pharmaceutical compounds were generated, which were found to be useful as diagnostic agents in vivo. The yields were calculated as radiochemical yields (RCYs): these yields are measured from the values of decay-corrected radioactivity. This is analogous to the concept of a regular yield but calculated for the radionuclide. Technically, the RCY is connected to the quantity of radioactivity in the product in percent compared to the initial value.

#### C–H amination

**Scheme 26:**
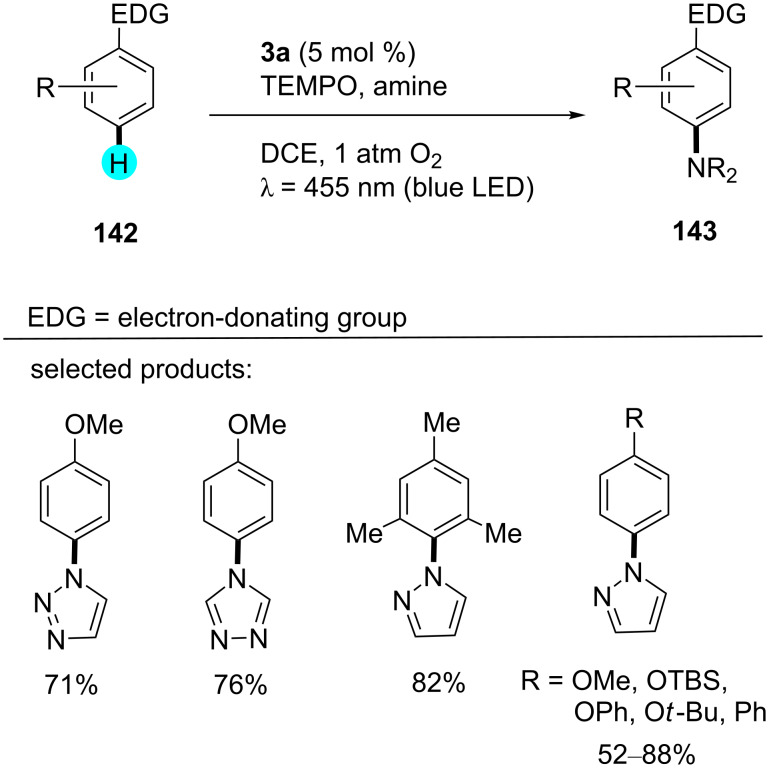
Aerobic amination with acridinium catalyst **3a**.

**Figure 24:**
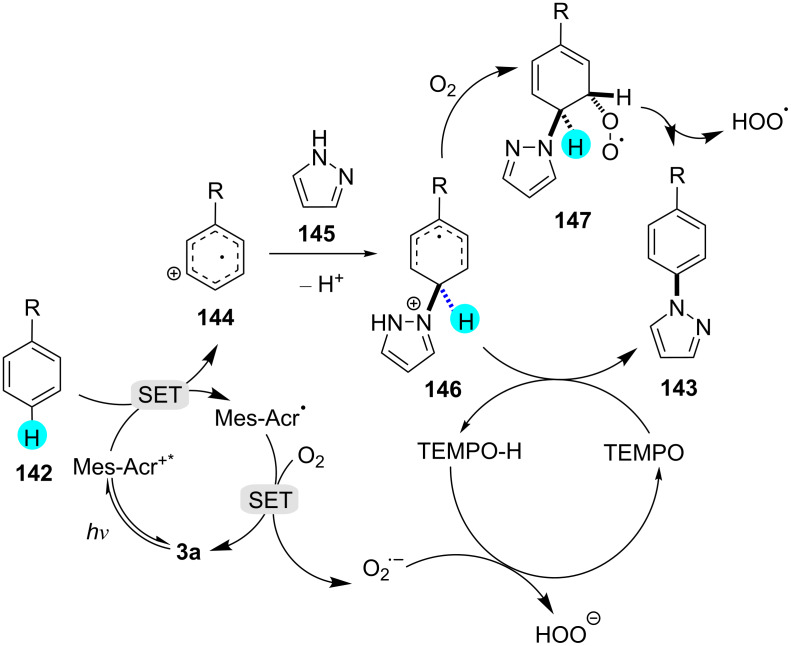
Plausible mechanism for the aerobic amination using acridinium catalyst **3a**.

**Aerobic aminations by acridinium catalysis:** In 2015, Nicewicz and his team established a method for arene aminations using photocatalyst **3a**, 3,6-di-*tert*-butyl-9-mesityl-10-phenylacridinium tetrafluoroborate, to generate a library of compounds in good to excellent yields (Scheme 26) [170]. They also exploited the photoredox catalyst **3b**, 3,6-di-*tert*-butyl-9-mesityl-10-phenylacridinim perchlorate, to carry out the reaction, but photocatalyst **3a** provided excellent yields. The proposed mechanism for these aminations is shown in Figure 24.

**Scheme 27:**
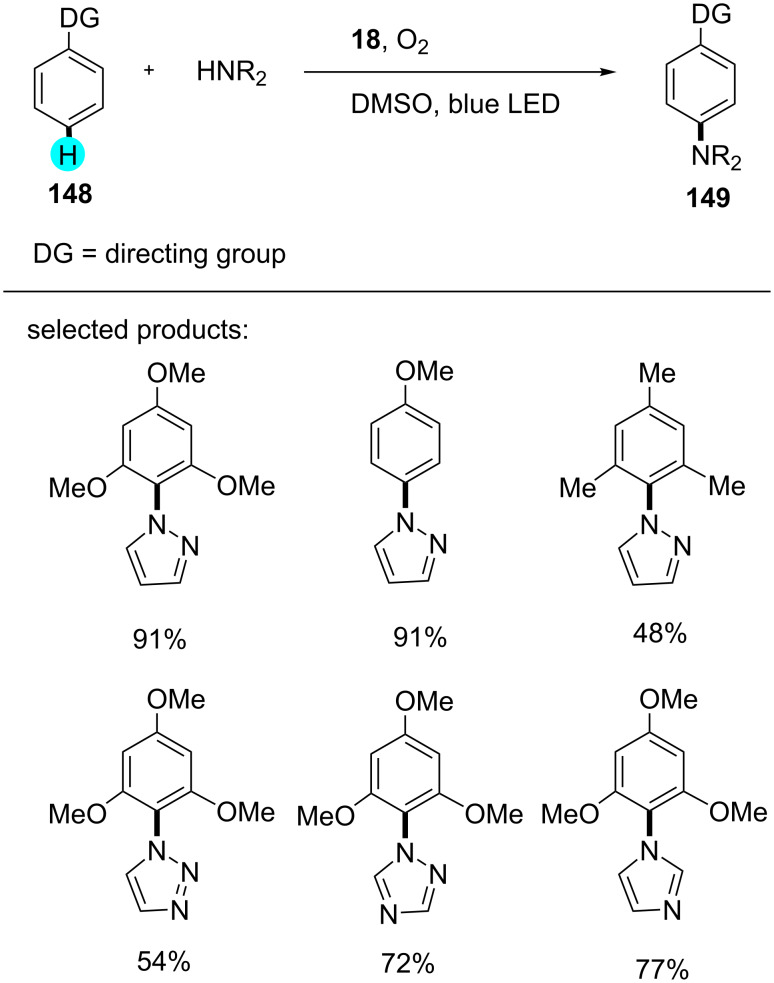
Aerobic aminations with semiconductor photoredox catalyst **18**.

**Aerobic aminations with semiconductor photoredox catalysts:** Semiconductor photoredox catalyst (SPC) **18** has proven to be an efficient catalyst that is thought to be economical, readily available, highly stable under the reaction conditions, and it was found to exhibit a balanced band gap (ca. 5.5 eV) [171–173]. In this context, quite recently, Wang’s group reported aerobic aminations with high selectivity using semiconductor photoredox catalyst **18** in the presence of molecular oxygen (Scheme 27) [174]. They discovered that the use of this SPC for C–H functionalizations provided high selectivity, sustainability, and environmentally friendly bond constructions.

#### C–H fluoroalkylation of arenes

**Scheme 28:**
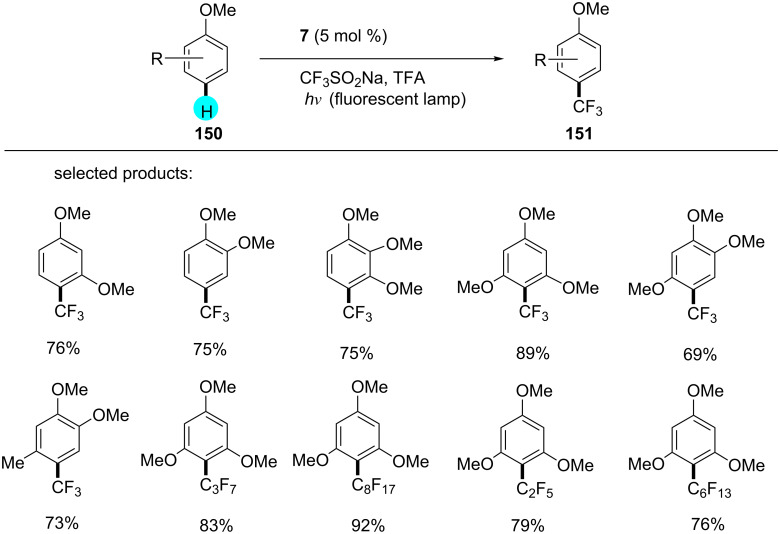
Perfluoroalkylation of arenes.

Fluoroalkylations in earlier reported methods required prefunctionalization of arenes, directing groups, etc. [175–179]. In this context, researchers were trying to find alternatives to the reported procedures. This being the case, in 2013, Itoh and co-workers reported the C–H perfluoroalkylation with photoredox catalyst **7** (Scheme 28), and they obtained the best yields with electron-rich arenes compared to electron-deficient ones [180].

#### C–H cyanation: synthesis of benzonitrile

**Scheme 29:**
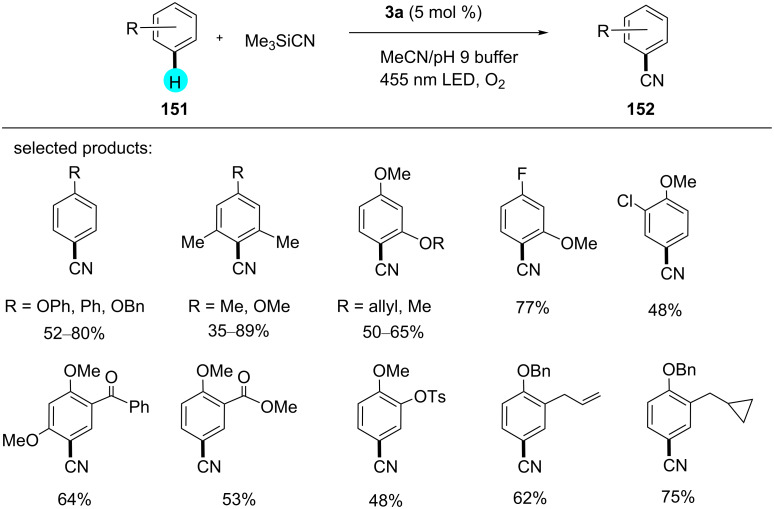
Synthesis of benzonitriles in the presence of **3a**.

**Figure 25:**
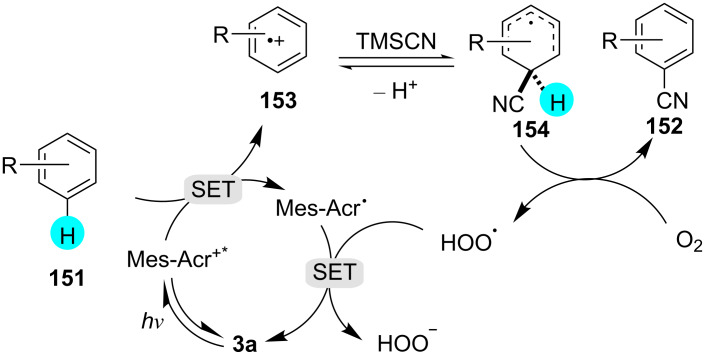
Plausible mechanism for the synthesis of substituted benzonitrile derivatives in the presence of **3a**.

Classically, benzonitrile was synthesized via Sandmeyer reaction, Rh/Co catalytic systems, or electrophilic reactions, and such reactions suffered from poor site selectivity. Therefore, utilizing the versatility of cyanoarenes, recently, Nicewicz and his team reported the preparation of cyanated products using the efficient photoredox catalyst **3a** at room temperature [181]. The reaction was compatible with the presence of electron-donating as well as electron-withdrawing groups, with TMSCN as an ideal cyanation reagent (Scheme 29). In the absence of light or a photocatalyst, no product was obtained. A plausible reaction mechanism involves the excitation of the photocatalyst by blue light, oxidizing **151** to **153**, a radical cation. The nucleophilic attack by TMSCN gives cyclohexadienyl radical **154**, which is oxidized by molecular oxygen to give the desired product **152** (Figure 25).

## Conclusion

C–H bond functionalizations via photoredox catalysis have proven to be unmatched by any other method in synthetic organic chemistry for the generation of intricate molecules. In this review, we highlighted site-selective *ortho* and *para* C–H bond functionalizations using photoredox catalysts. The versatile properties of these catalysts, such as low toxicity, their functioning at room temperature, and smooth irradiation requirements with low-energy lights (e.g., LEDs, fluorescent bulbs, etc.) made this area of research very interesting. Within a very short time, photoredox catalysis has emerged as an important future direction for modern synthetic chemistry, and we believe that the application in natural product synthesis and *meta* functionalization is highly desirable. In addition to this, to provide high selectivities and to allow for the combination of dual photoredox catalysis with HAT, the discovery of more effective and cheaper photoredox catalysts is of pressing need. Hopefully, the readers will utilize this collection of examples that has been presented in the light of photoredox catalysis.
